# Plasma Metabolomics Implicates Modified Transfer RNAs and Altered Bioenergetics in the Outcomes of Pulmonary Arterial Hypertension

**DOI:** 10.1161/CIRCULATIONAHA.116.024602

**Published:** 2017-01-30

**Authors:** Christopher J. Rhodes, Pavandeep Ghataorhe, John Wharton, Kevin C. Rue-Albrecht, Charaka Hadinnapola, Geoffrey Watson, Marta Bleda, Matthias Haimel, Gerry Coghlan, Paul A. Corris, Luke S. Howard, David G. Kiely, Andrew J. Peacock, Joanna Pepke-Zaba, Mark R. Toshner, S. John Wort, J. Simon R. Gibbs, Allan Lawrie, Stefan Gräf, Nicholas W. Morrell, Martin R. Wilkins

**Affiliations:** From the Department of Medicine, Imperial College London, Hammersmith Campus, United Kingdom (C.J.R., P.G., J.W., K.C.R.-A., G.W., M.R.W.); Department of Medicine, University of Cambridge School of Clinical Medicine, United Kingdom (C.H., M.B., M.H., M.R.T., S.G., N.W.M.); Cardiology Department, Royal Free Hospital, London, United Kingdom (G.C.); Institute of Cellular Medicine, Newcastle University and the Newcastle Upon Tyne Hospitals NHS Foundation Trust, United Kingdom (P.A.C.); National Pulmonary Hypertension Service, Imperial College Healthcare NHS Trust, Hammersmith Hospital, London, United Kingdom (L.S.H., J.S.R.G.); National Heart and Lung Institute, Imperial College London, Hammersmith Campus, United Kingdom (L.S.H., J.S.R.G.); Sheffield Pulmonary Vascular Disease Unit, Royal Hallamshire Hospital, United Kingdom (D.G.K.); Department of Infection, Immunity & Cardiovascular Disease, University of Sheffield, United Kingdom (D.G.K., A.L.); Scottish Pulmonary Vascular Unit, Golden Jubilee National Hospital, Glasgow, United Kingdom (A.J.P.); Pulmonary Vascular Disease Unit, Papworth Hospital, Cambridge, United Kingdom (J.P.Z., M.R.T.); Pulmonary Hypertension Service, Royal Brompton Hospital, London, United Kingdom (S.J.W.); and Department of Haematology, University of Cambridge, United Kingdom (S.G.).

**Keywords:** hypertension, pulmonary, metabolism, metabolome, metabolomics, pulmonary circulation

## Abstract

**Background:**

Pulmonary arterial hypertension (PAH) is a heterogeneous disorder with high mortality.

**Methods:**

We conducted a comprehensive study of plasma metabolites using ultraperformance liquid chromatography mass spectrometry to identify patients at high risk of early death, to identify patients who respond well to treatment, and to provide novel molecular insights into disease pathogenesis.

**Results:**

Fifty-three circulating metabolites distinguished well-phenotyped patients with idiopathic or heritable PAH (n=365) from healthy control subjects (n=121) after correction for multiple testing (*P*<7.3e-5) and confounding factors, including drug therapy, and renal and hepatic impairment. A subset of 20 of 53 metabolites also discriminated patients with PAH from disease control subjects (symptomatic patients without pulmonary hypertension, n=139). Sixty-two metabolites were prognostic in PAH, with 36 of 62 independent of established prognostic markers. Increased levels of tRNA-specific modified nucleosides (N2,N2-dimethylguanosine, N1-methylinosine), tricarboxylic acid cycle intermediates (malate, fumarate), glutamate, fatty acid acylcarnitines, tryptophan, and polyamine metabolites and decreased levels of steroids, sphingomyelins, and phosphatidylcholines distinguished patients from control subjects. The largest differences correlated with increased risk of death, and correction of several metabolites over time was associated with a better outcome. Patients who responded to calcium channel blocker therapy had metabolic profiles similar to those of healthy control subjects.

**Conclusions:**

Metabolic profiles in PAH are strongly related to survival and should be considered part of the deep phenotypic characterization of this disease. Our results support the investigation of targeted therapeutic strategies that seek to address the alterations in translational regulation and energy metabolism that characterize these patients.

## Abstract 2

Supplemental Digital Content is available in the text.

Pulmonary arterial hypertension (PAH) is a progressive vascular disorder that leads to increased pulmonary vascular resistance, right ventricular dysfunction,^1^ and premature death.^2^ The pathogenesis of the vascular pathology is poorly understood.^3,4^ Several genetic mutations have been reported in hereditary and isolated idiopathic presentations of PAH, providing insight into perturbed signaling pathways,^5,6^ and genome sequencing of clinically well-characterized patient cohorts is underway in anticipation of finding new mutations. A complementary approach to identifying the molecular drivers of PAH is to conduct deep molecular phenotyping of patients beyond standard clinical tests.

Metabolomic technologies such as ultraperformance liquid chromatography mass spectrometry enable the detection and semiquantitative measurement of hundreds of unique metabolites, representing a broad range of metabolic pathways, in small volumes of biofluids.^7^ These approaches have identified differences in circulating metabolites that distinguish physiological and disease states such as diabetes mellitus and systemic cardiovascular disorders and predict clinical outcomes.^8–10^ So far, few metabolomics studies have been undertaken in patients with pulmonary vascular disease. Evidence of abnormal oxidation, arginine, and sphingosine pathways has been found from mass spectrometry analysis of lung tissue from patients with PAH,^11,12^ and analysis of breath samples showed that exhaled volatile compounds discriminate between patients with severe idiopathic PAH and healthy volunteers.^13^ A targeted analysis of 105 circulating plasma metabolites in PAH, primarily amino acids, nucleosides, and their derivatives, showed that abnormal levels of tryptophan, purine, and tricarboxylic acid (TCA) cycle metabolites correlated to hemodynamic measures.^14^

In this study, we used a broad metabolomics platform to analyze 1416 metabolites in plasma from patients with idiopathic or heritable PAH (n=365) in 3 distinct cohorts and compared circulating levels with both healthy (n=121) and disease (n=139) control subjects. We identified specific metabolites that both discriminate patients with PAH from healthy and disease control subjects and independently predict survival. These metabolites included several modified nucleosides specific to transfer RNAs that indicate alterations in cell proliferation and translation of disease-related proteins, as well as several constituents of energy metabolism.

## Methods

### Sample Collection

Samples were obtained from patients with idiopathic or heritable PAH attending the National Pulmonary Hypertension Service at Hammersmith Hospital, London, between 2002 and 2015 and from patients recruited from other UK national centers as part of the National Cohort Study of Idiopathic and Heritable Pulmonary Arterial Hypertension (ClinicalTrials.gov. Unique identifier: NCT01907295). Control plasma samples were obtained from healthy subjects and disease control subjects, the latter being symptomatic patients presenting to the service but in whom pulmonary hypertension was excluded by cardiac catheterization. The diagnosis of PAH was based on standard criteria from the most recent guidelines.^1^ Vasoresponders were defined as those whose mean pulmonary artery pressure dropped >10 mm Hg to <40 mm Hg with preserved cardiac output in response to an acute pulmonary vasodilator challenge and remained stable on calcium channel blocker therapy alone for at least 1 year.^1^ Whole-genome sequencing data from the UK National Institute of Health Research BRIDGE consortium (Biomedical Research Centres Inherited Diseases Genetic Evaluation) were used to determine which patients had known pathogenic mutations in the gene encoding the bone morphogenetic protein type II receptor (*BMPR2*).^5^

Venous blood samples were drawn from the antecubital fossa and collected in EDTA Vacutainer tubes (BD, Oxford, UK), immediately put on ice, centrifuged (1300*g*, 15 minutes) within 30 minutes, and stored at −80°C until required. World Health Organization functional class and 6-minute walk distance at the sample date and clinical biochemical data (within 30 days) were recorded. All subjects provided informed written consent, and local research ethics committees approved the study. A subset of patients consented to provide additional samples at later dates while attending follow-up clinical appointments.

### Metabolomics

Metabolomic profiling by ultraperformance liquid chromatography mass spectrometry was conducted by Metabolon (Durham, NC),^7^ which provided semiquantitative assessment of 949 named and 467 unnamed metabolite levels, annotated with pathways. Named compounds identified from mass and fragmentation analysis but yet to be confirmed with standards are indicated by asterisks. Details can be found in the online-only Data Supplement.

### Angiogenin

Plasma angiogenin levels were determined by ELISA (reference No. DAN00, R&D Systems, Abingdon, UK) per the manufacturer’s guidelines, with EDTA plasma diluted 1:800 before assay.

### Statistical Analysis

To prevent skewing of results by outliers, initial group comparisons between control subjects and patients were performed with nonparametric Mann Whitney *U* tests. Before modeling, metabolites with distributions that were not normal were either transformed by log_10_ or power transformations (x^Y^, with Y from −2 to 2 in 0.5 steps, as performed for Box-Cox transformations^15^), whichever best normalized the data on the basis of Kolmogorov-Smirnov tests, or ranked if no test met *P*>0.05. Samples for which metabolites were undetected were imputed with the minimum detected level for the metabolite. Data in all groups were *z* score transformed on the basis of the mean and standard deviation in all healthy control subjects for ease of comparisons. Data are presented as absolute numbers, percentages, or mean or median (±SD) and percentile range.

Linear regression analysis was conducted to assess the relationships between metabolite levels, diagnoses, and potential confounders to determine whether differences in metabolite levels between groups were independent of age, sex, ethnicity, body mass index, drugs, and renal and hepatic dysfunction. In the disease control and PAH cohorts, preserved renal function was defined as creatinine <75 µmol/L, and preserved liver function was defined as bilirubin <21 µmol/L. Logistic regression was conducted to determine metabolites that independently distinguished between diagnostic groups. Orthogonal partial least squares discriminant analysis modeling was used to test the performance of these metabolites. *R*^2^ scores indicate model performance, and Q^2^ scores estimate reproducibility on the basis of cross-validation (subjects were divided into 7 groups, and their diagnosis was predicted on the basis of the other subjects in 7 analyses). Pathway enrichment analysis was conducted on discriminating and prognostic metabolites with the Fisher’s exact test.

All survival analyses were performed with the use of time from sampling to death/census. Cox regression analysis was used to identify prognostic predictors, with proportional hazard assumptions tested and Kaplan-Meier plots used to illustrate events from time of sampling in relation to metabolite levels. Receiver-operating characteristic curves were used to assess discriminating and prognostic values of metabolites against diagnosis and all-cause mortality, respectively. Hierarchical clustering on the basis of euclidean distances was used to assess whether metabolites and patients clustered by functional pathways and phenotypes, respectively.

Network analysis was performed by calculating second-order Spearman rank correlations with ParCorA^16,17^ and visualized with Cytoscape.^18^ “Hub” nodes are metabolites with the most “edges” (correlations) to other metabolites.

Statistical analysis was performed with IBM SPSS Statistics 22 (International Business Machines Corp, New York, NY), Matlab (Matrix Laboratory, MathWorks, Natick, MA), Microsoft Excel (Microsoft, Redmond, WA), SIMCA-P software, (Umetrics, Umea, Sweden), and R with RStudio and associated packages.^19^

## Results

### Metabolites Distinguishing Between PAH and Control Subjects

We first compared plasma metabolite profiles from 116 consecutive patients with idiopathic or heritable PAH attending Hammersmith Hospital between November 2011 and August 2013 and 58 healthy control subjects (Table 1). To minimize confounding factors, only patients with PAH who were 19 to 70 years of age were compared with age- and sex-matched healthy control subjects in this analysis. Results were validated in 75 patients with PAH recruited between 2002 and 2015 against a separate control group (n=63). A second validation analysis used 174 patients with PAH recruited from other specialist centers in the United Kingdom from August 2013 to June 2015 and compared with all control subjects. Metabolites identified as xenobiotics or detected in <95% of samples were excluded from the analysis, leaving 686 well-quantified biological metabolites.

**Table.**
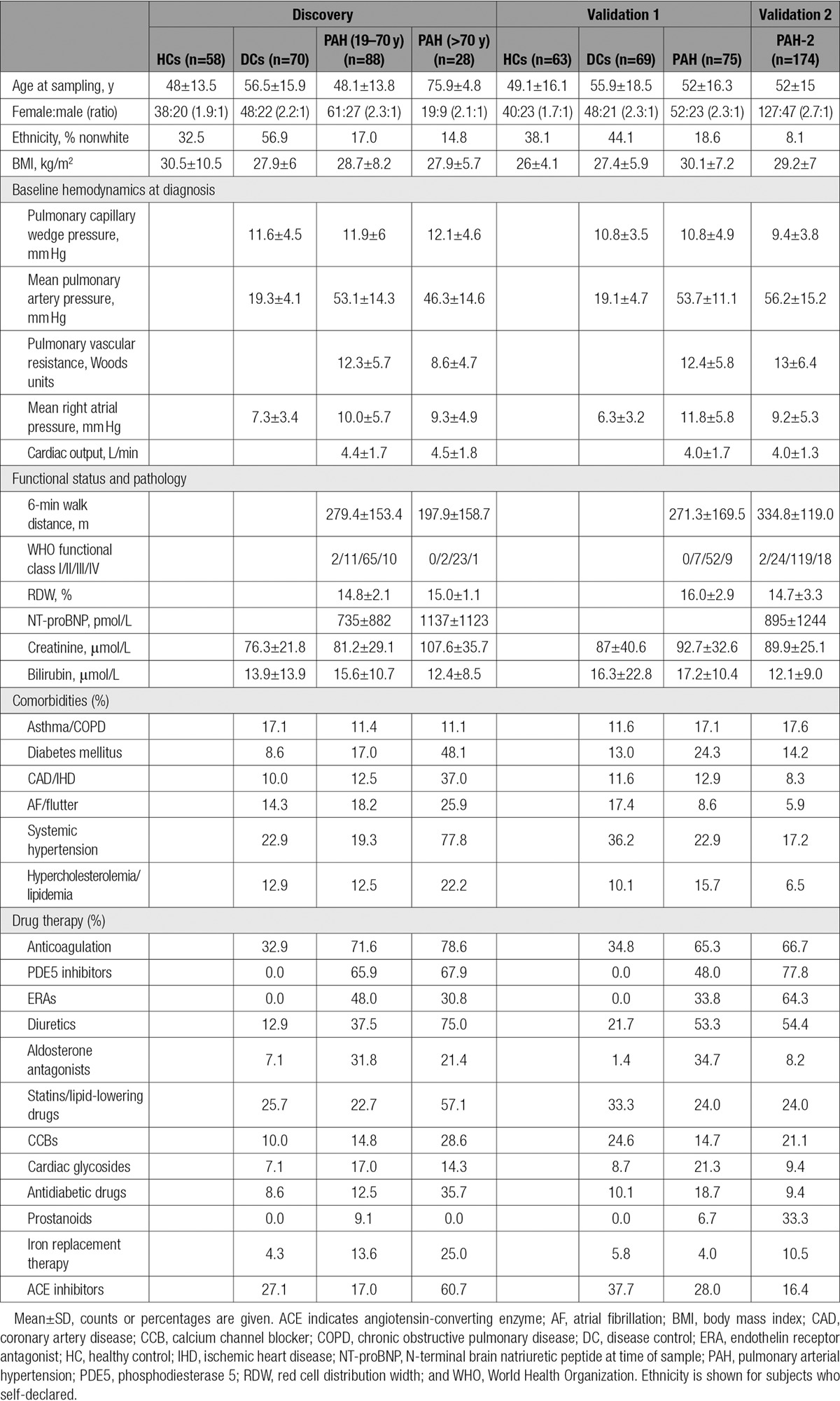
Cohort Characteristics

**Figure 1.**
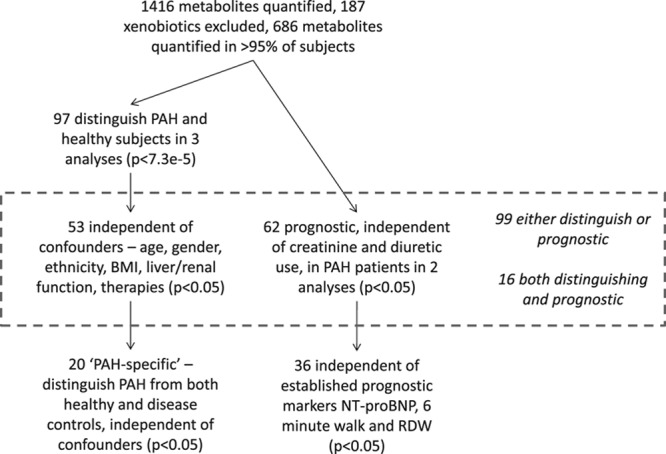
**Analysis flow chart.** Summary of analytic workflow showing numbers of metabolites that distinguish patients with pulmonary arterial hypertension (PAH) from control subjects and/or are prognostic in PAH. NT-proBNP indicates N-terminal brain natriuretic peptide; and RDW, red cell distribution width.

Circulating levels of 97 metabolites distinguished PAH from healthy subjects in all 3 analyses after Bonferroni correction (*P*<7.3e-5). Of these metabolites, 53 distinguished healthy control and PAH subjects after correction for potential confounders, including age, sex, ethnicity, body mass index, creatinine, bilirubin, and drug therapies (*P*<0.05; Table I in the online-only Data Supplement and Figure 1). The most common confounders associated with metabolite levels were liver function (bilirubin) and renal function (creatinine). To determine whether these metabolite differences could be detected before the initiation of PAH therapies, we performed a subanalysis comparing 40 patients who were treatment naïve at the time of sampling, and all 53 metabolites distinguished this group from healthy control subjects (*P*<0.05; Table I in the online-only Data Supplement). Patients with pathogenic *BMPR2* mutations (n=42) had metabolite levels similar to PAH patients without these mutations (Figure I and Table I in the online-only Data Supplement).

**Figure 2.**
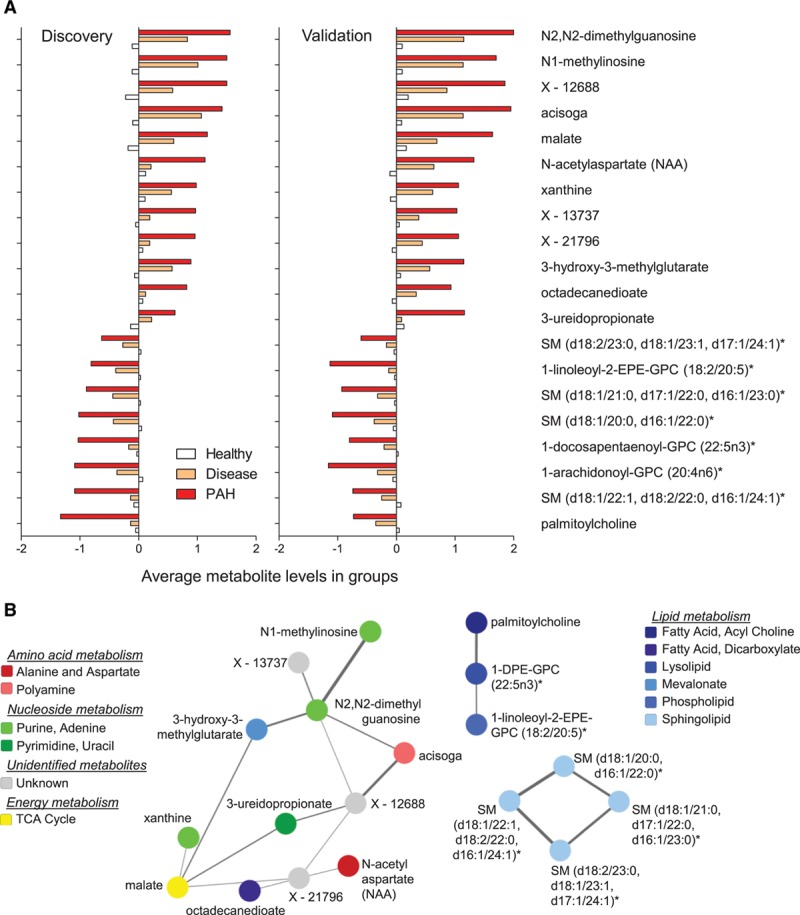
**Metabolites that discriminate patients with pulmonary arterial hypertension pulmonary arterial hypertension (PAH) from control subjects. A**, Average metabolite levels in PAH and control subjects for 20 metabolites found to significantly distinguish patients with PAH from both healthy and disease control subjects independently of potential confounders. Values plotted are *z* scores calculated from the mean and standard deviation of all healthy volunteers in the study. Negative values indicate metabolites at lower levels in patients vs healthy control subjects, and positive values indicate higher levels of metabolites in patients. For the discovery analysis, only data from patients with PAH who were 19 to 70 years of age are plotted; for the validation analysis, all patients data are shown. **B**, Network analysis of the same 20 metabolites on the basis of second-order correlations. Line thickness indicates strength of correlations (all *P*<0.0001). *Probable metabolite identity but unconfirmed (see Methods). DHE indicates docosahexaenoyl; DHEA-S, dehydroisoandrosterone sulfate; DPE, docosapentaenoyl; EPE, eicosapentaenoyl; GPC, glycerophosphocholine; and SM, sphingomyelin.

Given that many metabolic alterations might occur in a chronic disease such as PAH, we set out to prioritize more disease-specific metabolites by comparing the patients with PAH with disease control subjects, the latter comprising symptomatic patients in whom pulmonary hypertension had been excluded. We again adopted a discovery-and-validation design with 2 groups of disease control subjects (n=70 and 69). A subset (20 of 53) of the metabolites distinguished patients with PAH from disease control subjects after correction for potential confounders (*P*<0.05; Table I in the online-only Data Supplement). These “PAH-specific” differences in metabolites included increases in purine, polyamine, and TCA cycle metabolites and decreases in phosphocholines and sphingomyelins (Figure 2A), with network analysis showing the importance of hub metabolites N2,N2-dimethylguanosine and malate (Figure 2B).

### Discriminant Analyses to Distinguish PAH and Control Groups

**Figure 3.**
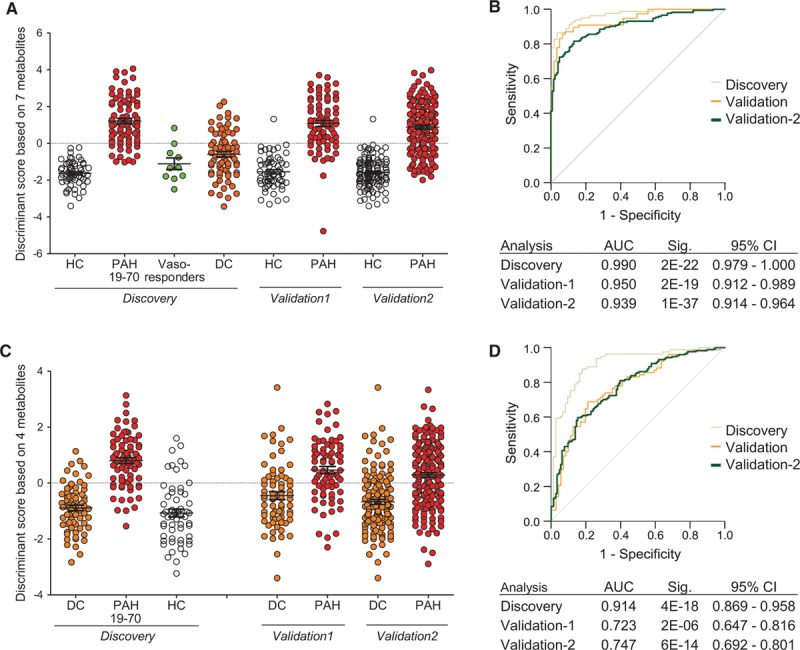
**Discriminant analysis models on the basis of low numbers of metabolites distinguish patients with pulmonary arterial hypertension (PAH) from control subjects. A** and **C**, Dot plots showing individual subjects’ model scores for healthy control subjects (HC), patients with PAH, vasoresponders, and disease control subjects (DC) in discovery and validation analyses. Metabolites were selected by logistic regression of PAH-HC (**A**) and PAH-DC (**C**) comparisons. **B** and **D**, Receiver-operating characteristic curves showing the performance of models in distinguishing PAH from HC (**B**) and DC (**D**) subjects. AUC indicates area under the curve; and CI, confidence interval.

To identify a minimal set of metabolites that could in combination best distinguish patients with PAH, we performed logistic regression analysis. We found that 7 of 53 metabolites—dehydroisoandrosterone sulfate (DHEA-S), methionine sulfone, N1-methylinosine, oleoylcarnitine, palmitoylcholine, sphingomyelin (d18:1/20:0, d16:1/22:0)*, and X-24513—independently distinguished patients with PAH (19–70 years of age) and healthy subjects in the discovery analysis with 90% accuracy in an orthogonal partial least squares discriminant analysis (*R*^2^=0.64, Q^2^=0.61). This model classified healthy subjects and patients with PAH in the 2 validation analyses with 89% and 84% accuracy, respectively. In addition, 90% (9 of 10) of PAH vasoresponders in the discovery cohort had metabolite levels typical of healthy control subjects (Figure 3A and 3B).

Four of the 20 PAH-specific metabolites—N-acetylaspartate, octadecanedioate, palmitoylcholine, and X-13737—distinguished patients with PAH and disease control subjects with 83% accuracy in the discovery analysis (*R*^2^=0.49, Q^2^=0.47). This model classified disease control subjects and patients with PAH in the 2 validation analyses with 69% and 67% accuracy, respectively (Figure 3C and 3D).

### Survival Analysis of Plasma Metabolites of Interest in PAH

We hypothesized that metabolites most closely related to the disease pathobiology would be associated with clinical outcomes. To identify metabolites associated with disease progression and mortality, we performed survival analyses. Twenty-eight of 116 and 25 of 75 patients died in the discovery and first validation PAH groups with an average follow-up of 3.3±1.0 and 4.5±4.0 years, respectively. The length of patient follow-up in the second validation cohort was insufficient to permit analysis. After correcting for creatinine and diuretic use, no other potentially confounding factor was associated with survival. Of the 686 well-quantified metabolites, 640 met the assumptions of Cox regression analysis, and 62 of these were prognostic after accounting for creatinine and diuretic use in both analyses. Receiver-operating characteristic analysis at 3 years of follow-up confirmed that these metabolites were prognostic and identified optimal cutoffs (*P*<0.05; Figure II in the online-only Data Supplement).

**Figure 4.**
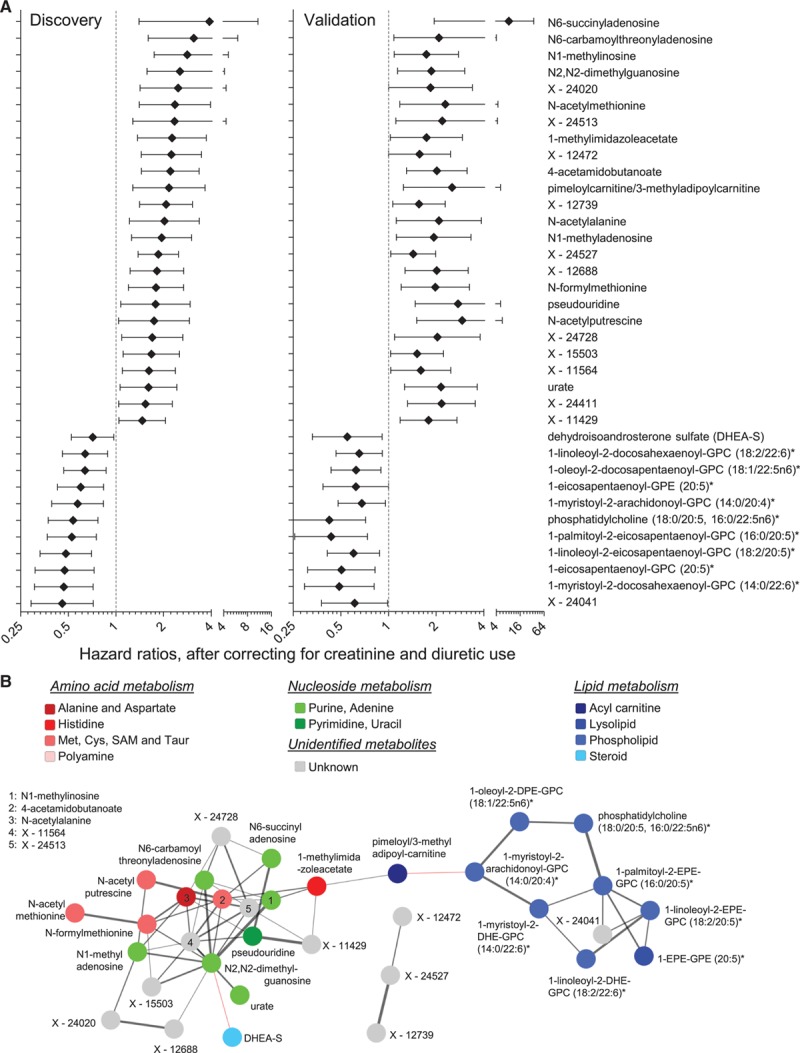
**Prognostic metabolites independent of established risk factors. A**, Hazard ratios after correction for creatinine and diuretic use of 36 metabolites that were prognostic in patients with pulmonary arterial hypertension independently of red cell distribution width, N-terminal brain natriuretic peptide, and 6-minute walk distance. Hazard ratios indicate the risk of a change in each metabolite of 1 SD for ease of comparison. Patients of all ages were included in both the discovery and validation survival analyses. **B**, Network analysis of the same 36 metabolites on the basis of second-order correlations. Line thickness indicates strength of correlations (all *P*<0.0001). Red lines indicate negative correlations. *Probable metabolite identity but unconfirmed (see Methods). DHE indicates docosahexaenoyl; DHEA-S, dehydroisoandrosterone sulfate; DPE, docosapentaenoyl; EPE, eicosapentaenoyl; GPC, glycerophosphocholine; GPE, glycerophosphoethanolamine; Met, Cys, SAM and Taur, methionine, cysteine, S-adenosylmethionine and taurine; and SM, sphingomyelin.

To identify metabolites that report on novel pathways independently of current prognostic estimates, we compared the 62 prognostic metabolites with 3 markers previously found to best predict survival in our patients, namely N-terminal brain natriuretic peptide, 6-minute walk distance, and red cell distribution width.^20^ Thirty-six of 62 of the metabolites were independent of these measures (*P*<0.05; Figure 4A and Table II in the online-only Data Supplement), and network analysis indicated 2 main clusters with hub metabolites, including again, among others, N2,N2-dimethylguanosine (Figure 4B).

### Enrichment and Clustering of Metabolites of Interest

**Figure 5.**
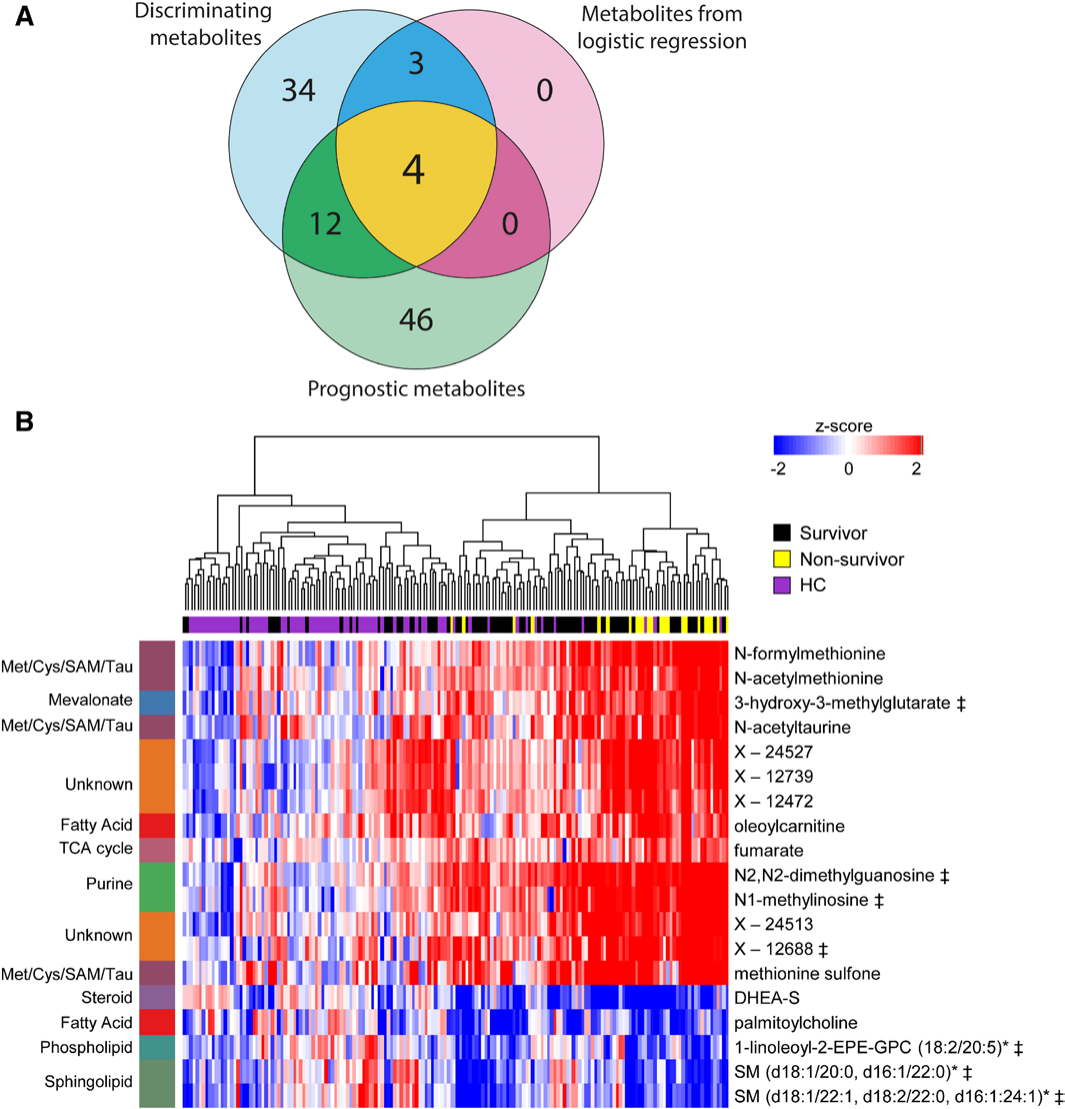
**Hierarchical clustering of 19 discriminating and prognostic metabolites in patients with pulmonary arterial hypertension (PAH). A**, Venn diagram shows overlap between metabolites that discriminate patients with PAH from healthy control subjects in all 3 cohorts from logistic regression between patients with PAH and healthy control subjects and prognostic metabolites in the discovery and first validation cohorts. **B**, Clustering of the 19 overlapping metabolites from **A** is shown between healthy control subjects (HC; n=58), PAH survivors (n=110, alive at 3 years after sample), and nonsurvivors (n=24) in the discovery analysis. Red indicates metabolite levels that are increased (and blue levels that are decreased) in patients with PAH vs control subjects. *Probable metabolite identity but unconfirmed (see Methods). ‡Metabolites also distinguish PAH from disease control subjects. DHEA-S indicates dehydroisoandrosterone sulphate; EPE, eicosapentaenoyl; GPC, glycerophosphocholine; and SM, sphingomyelin.

The above analyses identified and validated, after controlling for confounders, a total of 99 metabolites that were either discriminating or prognostic in PAH, representing 25 metabolic pathways. Six pathways in particular were enriched with metabolites of interest, including fatty acid (acyl carnitines), polyamine, and nucleoside metabolism (*P*<0.05; Table III in the online-only Data Supplement). Sixteen of these metabolites both discriminated PAH and were prognostic; these, along with the 3 other metabolites selected by logistic regression modeling to best distinguish patients with PAH and healthy subjects, clustered into defined metabolic pathways (Figure 5).

### Analysis of Serial Samples

Changes in metabolite levels in individuals over time may indicate pathways that report clinical improvement or the correction of which leads to improved outcomes. We analyzed serial samples from 86 patients who were followed up for a minimum of 1 year (median, 1.50 years; interquartile range, 1.33–2.95 years) after the second sample. Twenty-nine patients died during follow-up. Changes in metabolite levels between the 2 samples (median time between samples, 1.75 years; interquartile range, 1.07–2.58 years) were compared between survivors and nonsurvivors.

**Figure 6.**
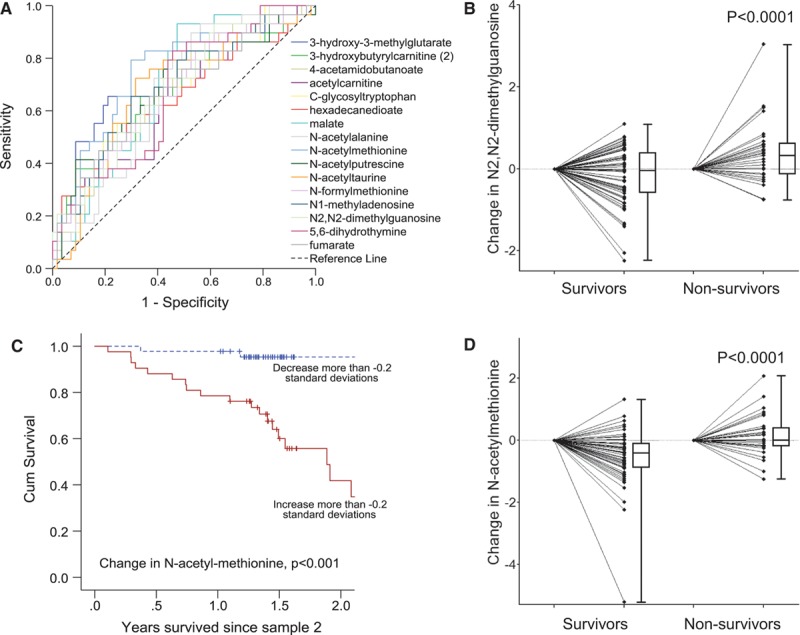
**Analysis of serial samples. A**, Receiver-operating characteristic analysis of changes in metabolite levels and survival status at last follow-up. **B** and **D**, Changes in individual patient metabolite levels grouped by survival status at last follow-up. **C**, Kaplan-Meier analysis illustrating survival over time in patients with pulmonary arterial hypertension divided into groups according to the changes in N-acetyl-methionine levels between serial samples.

Changes in 27 of 99 metabolites, including several modified amino acids and nucleosides, were significantly different between survivors and nonsurvivors (*P*<0.05). Receiver-operating characteristic analysis confirmed these associations (Table IV in the online-only Data Supplement) and identified prognostic cut-offs (Figure 6).

### Association of Elevated Modified Nucleosides With Elevated Plasma Angiogenin

**Figure 7.**
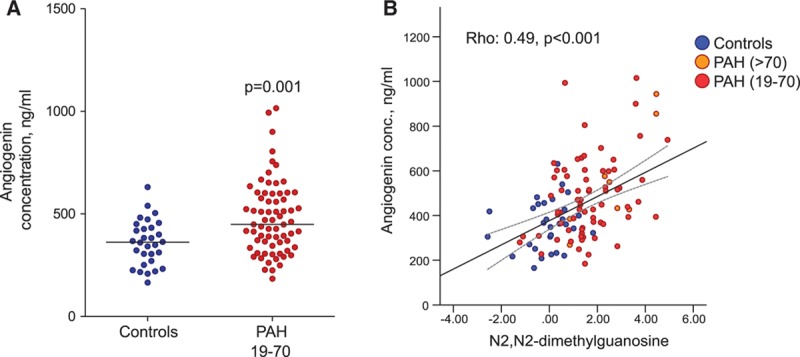
**Circulating angiogenin levels. A**, Plasma angiogenin levels determined by ELISA in healthy control subjects and patients with PAH. **B**, Scatterplot of plasma N2,N2-dimethylguanosine vs plasma angiogenin in control subjects and patients with pulmonary arterial hypertension (PAH). Statistics shown are from the Spearman rank test.

Modified nucleosides can be released into the circulation during stress after cleavage of transfer RNAs (tRNAs) by the ribonuclease angiogenin.^21^ To determine whether this mechanism was relevant to PAH, we measured plasma angiogenin in a representative subset of age- and sex-matched healthy control subjects and patients with PAH from the discovery analysis (Table V in the online-only Data Supplement). Angiogenin levels were elevated in plasma from patients with PAH and correlated with N2,N2-dimethylguanosine levels (ρ=0.49, *P*<0.001; Figure 7). The strength of correlation was similar in male and female subjects (data not shown).

## Discussion

This study represents the most comprehensive analysis of circulating metabolites in patients with PAH to date. It is the first to robustly identify and validate differences in comparison to both healthy and symptomatic disease control subjects without pulmonary hypertension and to associate metabolic profiles with outcomes in PAH, strengthening the evidence that the pathways identified could be important modifiers of disease progression. Changes in the levels of metabolites over time were associated with survival in a direction that suggests that correction of these disturbances is linked to improved outcomes. In agreement with this, patients defined as vasoresponders, who have excellent outcomes on calcium channel blocker therapies, demonstrated metabolic profiles more similar to those of healthy control subjects than to other patients. Metabolic profiles seen in incident cases were similar to those with established PAH, emphasizing that metabolic dysregulation is not corrected in the majority of cases by current therapy.

### Modified Nucleosides

Two of the most robust distinguishing and prognostic differences identified in patients with PAH were increased levels of N1-methylinosine and N2,N2-dimethylguanosine. These are recognized epigenetic, posttranscriptional modifications of tRNA,^21–23^ and other tRNA modifications also found to be increased and prognostic included pseudouridine, N6-carbamoylthreonyladenosine and N1-methyladenosine. N2,N2-dimethylguanosine is found in the majority of tRNAs at position 26, upstream of the anticodon sequence at positions 34 to 36, and promotes the folding of tRNAs toward the classical cloverleaf structure.^24^ N1-methylinosine is found 3′ adjacent to the anticodon at position 37 of eukaryotic tRNAs and is formed from inosine by a specific S-adenosylmethionine–dependent methylase.^25^ Increased serum and urine levels of N2,N2-dimethylguanosine, as well as pseudouridine and 1-methylinosine, have been observed in multiple solid tumor malignancies^26^ and may reflect the general upregulation of the translational apparatus, including tRNA turnover, in hyperproliferative cancerous cells.^27^ Increased circulating 1-methyladenosine has also been shown to be an early indicator of oxidative stress, cell damage, and mortality in kidney disease.^28^

Intracellular tRNA pools are dynamically regulated. For example, under stress, tRNAs required for the translation of stress response proteins are preferentially expressed.^21^ The altered levels of specific nucleoside modifications in patients with PAH may reflect preferential expression of the tRNAs that harbor them as part of a switch toward translation of disease-related proteins. In addition, stress-induced cleavage of tRNA produces fragments that propagate the stress response and interfere with eukaryotic initiation factors-4G and -4F.^21^ Furthermore, eukaryotic initiation factor-2α kinase-4 (GCN2), which prevents eukaryotic initiation factor-2α from interacting with the initiating Met-tRNA, suppressing general protein synthesis and activating stress-inducible transcription factors, is mutated and causally implicated in some cases of pulmonary vascular disease.^29^ Mutations in tRNA genes themselves have also been reported to cause pulmonary hypertension driven by mitochondrial dysfunction.^30^

The initial cleavage of tRNAs is mediated by angiogenin,^21^ which we showed to be elevated in the plasma of patients with PAH in concert with elevated levels of modified nucleosides. Angiogenin is also upregulated in cancer cells, mediating angiogenesis, cell proliferation, and protection from apoptosis,^31^ and is increased in breath condensates from patients with pulmonary hypertension,^32^ indicating a possible pulmonary origin in this disease. Angioproliferative plexiform vascular lesions are characteristic of advanced PAH, and the proangiogenic activity of angiogenin is inhibited by mutation of its ribonuclease active site,^33^ suggesting that elevated angiogenin and nucleoside levels may report patients developing this type of pulmonary vascular remodeling. Alterations in tRNA biology appear to be capable of driving the development of rare forms of pulmonary hypertension and are closely linked to the progression of PAH. Circulating levels of modified nucleosides may reflect increases in both pulmonary vascular cell proliferation and stress.

### Energy Metabolism

Significant alterations were observed in several pathways related to cellular energy production, with accumulation of multiple acylcarnitines, glutamate, and TCA cycle intermediates. Their accumulation in patients with PAH may represent a failed attempt to increase utility of fatty acids as an energy source, perhaps reflecting the inability of fatty acid beta-oxidation to keep pace with the demands of the overburdened right ventricle. Glutaminolysis is another alternative energy production pathway to glucose oxidation, with the product glutamate entering the TCA cycle as α-ketoglutarate. Inhibition of glutaminolysis and restoration of glucose oxidation have beneficial effects in rat models of right ventricular hypertrophy.^34^ Increased circulating glutamate levels have previously been seen in cancer patients^35^; however, antiglutaminolysis therapeutic targets have demonstrated toxic side effects.^36^ The buildup of TCA intermediates and the precursors to the molecules that enter the cycle (acylcarnitines and glutamate) may indicate dysfunction of this cycle, or at least the inability to keep pace with the demands of the most active cells such as proliferating pulmonary vascular cells. Increased levels of citrate, succinate, and fatty acid metabolites have been demonstrated in lung tissue from patients with PAH,^11^ suggesting that dysfunctional energy metabolism is a feature of the diseased tissue. Restoration of glucose oxidation by dichloroacetate therapy is under investigation as a treatment for PAH (ClinicalTrials.gov. Unique identifier: NCT01083524),^37^ and maximizing the capacity of the TCA cycle to process the acetyl-CoA produced may be a complementary therapeutic approach.

Consistent with previous reports, we found a significant increase in the circulating levels of long-chain acylcarnitines (oleoylcarnitine)^38^ and short-chain (myristoylcarnitine, acetylcarnitine, hydroxbutyrylcarnitine) and medium-chain (adipoylcarnitine, suberoylcarnitine) products. The accumulation of acylcarnitines may itself be detrimental, effecting cardiac electrophysiological changes and arrhythmias.^39^ There is also increasing evidence that accumulation of long-chain acylcarnitines may contribute to insulin resistance,^40^ which is itself common and associated with prognosis in PAH.^41^

### Lipids, Steroids, Polyamines, and Tryptophan Metabolites

Multiple sphingomyelin and phosphatidylcholine lipid species were significantly reduced in patients with PAH, relating to increased mortality. Sphingomyelins are the most abundant subclass of sphingolipids, with other subclasses including sphinogosines, ceramides, and glycophospholipids.^42^ In patients with chronic obstructive pulmonary disease, low plasma levels of several sphingomyelins relate to disease severity.^43^ As a membrane constituent, sphingomyelins are implicated in transmembrane signaling and are generated from phosphatidylcholine and ceramide by sphingomyelin synthase, the knockout of which leads to mitochondrial dysfunction and reduced insulin release.^44^ Sphingomyelins may also be considered a source of ceramide, which directly (and indirectly through other active lipid products) regulates cell proliferation, apoptosis, cell migration, and autophagy.^45^

Reduced lineoyl-glycerophosphocholine has been shown to be an early marker of insulin resistance in nondiabetics,^46^ and decreased circulating levels of several phosphatidylcholines were seen in patients with severe heart failure.^47^ Phospholipids are also sources of multiple cellular signaling molecules, including eicosanoids such as prostacyclin,^48^ levels of which are known to be reduced in pulmonary hypertension, with replacement an established treatment option.

Circulating levels of DHEA-S and its metabolites (androsterone, epiandrosterone, and androstenediol/4-androsten-3β, 17β-diol disulfate) were reduced in patients with PAH compared with healthy control subjects, consistent with a recent report of reduced circulating levels of DHEA-S in a small cohort of 23 male patients with PAH compared with healthy control subjects.^49^ Differences in DHEA-S between patients with PAH and control subjects were independent of the more subtle effects of both sex and age (Figure III in the online-only Data Supplement), and lower DHEA-S levels were independently associated with mortality. Treatment with DHEA or DHEA-S has repeatedly been shown to prevent and reverse pulmonary hypertension in experimental rat models,^50^ with clinical trials ongoing in chronic obstructive pulmonary disease–associated pulmonary hypertension (ClinicalTrials.gov. Unique identifier: NCT00581087).

We found increased levels of a breakdown product of N1-acetylspermidine, acisoga. Other metabolites of polyamine metabolism (4-acetamidobutanoate and N-acetylputrescine) were increased in PAH in relation to bilirubin levels and were prognostic in 2 distinct PAH cohorts, independently of established prognostic markers. Several animal models of pulmonary hypertension have demonstrated evidence of increased polyamine levels and metabolism in lung tissue.^51^ Administration of monocrotaline to rats led to significantly increased levels of polyamines and the development of pulmonary hypertension and right ventricular hypertrophy, which could be prevented by the administration of an inhibitor of polyamine biosynthesis,^52^ suggesting that these molecules may be novel therapeutic targets.

In our study, we validated findings of elevated circulating tryptophan metabolites^14^ with increased C-glycosyltryptophan and kynurenine in patients with PAH compared with healthy control subjects, but changes in kynurenine were related to increased bilirubin levels and liver dysfunction. Levels of tryptophan and its other major metabolite, serotonin, were not significantly altered in our analysis.

### Limitations

The majority of patients included in this study were patients with prevalent disease on established treatments. A subanalysis conducted with 40 patients with incident disease showed similar results. Corrections were also made for potential treatment effects in the main analyses, including PAH-specific and comorbidity-related therapies, as well as demographics and renal/hepatic function.

Patients and control subjects were sampled in the nonfasting state, and information on insulin resistance was not available for all participants. Patients were also sampled from a peripheral vein. The stronger performance of discriminating models in discovery analyses suggests that optimization could further improve their performance in distinct cohorts. Strongly correlated metabolites would not have been selected in the discriminant modeling, so each metabolite used in the final models may represent clusters of multiple metabolites. Evidence of tissue specificity and the source of circulating metabolites in pulmonary hypertension require further studies, for example, by transpulmonary sampling and direct measurements from tissue samples, to better localize the source of informative metabolites. Plasma levels of metabolites may not reflect levels in the most important tissues; for example, increased bile acid metabolites have been demonstrated in PAH lung tissue,^53^ but no differences were seen in circulating levels in our study.

### Summary and Conclusions

Increased circulating modified nucleosides (N2,N2-dimethylguanosine, N1-methylinosine), TCA cycle intermediates (malate, fumarate), glutamate, fatty acid acylcarnitines, and polyamine metabolites and decreased levels of steroids, sphingomyelins, and phosphatidylcholines are characteristics of patients with PAH that distinguish them from symptomatic patients without pulmonary hypertension. Improvements in circulating metabolite levels are associated with a better prognosis and could be used to monitor response to PAH treatments. Indeed, our results support the investigation of therapeutic strategies targeted at alterations in energy metabolism in PAH and suggest options for correcting translational regulation that also merit further study.

## Acknowledgments

This article presents independent research that was supported by the National Institute for Health Research (NIHR)/Wellcome Trust Imperial Clinical Research Facility at Imperial College Healthcare NHS Trust, London, UK. The views expressed are those of the authors and not necessarily those of the NHS, the NIHR, or the Department of Health. The authors are indebted to Souad Ali and Sharon Meehan for blood sample collection and to Abdul Mulla, George Villas, Lavanya Ranganathan, and the TRIPHIC (Translational Research in Pulmonary Hypertension at Imperial College) system for the processing and pseudonymization of patient information. The authors also thank all the staff and participants of the BRIDGE (BioResource–Rare Diseases) and the National Cohort Study of Idiopathic and Heritable Pulmonary Arterial Hypertension for their invaluable and ongoing contributions.

## Sources of Funding

Dr Rhodes is supported by a British Heart Foundation (BHF) Intermediate Basic Science Research Fellowship (FS-15-59-31839) and Dr Lawrie by a BHF Senior Basic Science Fellowship (FS/13/48/30453). Dr Wilkins is supported by a BHF program grant (RG/10/16/28575). Dr Morrell is an NIHR senior investigator. This research was also supported by a BHF Special Project (SP/12/12/29836), MRC Experimental Challenge Award (MR/K020919/1), the NIHR Bioresource for Rare Diseases, Imperial College and Cambridge NIHR Biomedical Research Centres, and the NIHR Rare Diseases Translational Research Collaboration.

## Disclosures

None.

## Supplementary Material

## Clinical Perspective

### What Is New?

- This study provides a comprehensive analysis of circulating metabolite levels in patients with pulmonary arterial hypertension (PAH) and control subjects.
- It is the first study to relate metabolite levels to clinical outcomes in PAH.
- Increases in circulating modified nucleosides originating from transfer RNAs, energy metabolism intermediates, tryptophan, and polyamine metabolites and decreased steroids, sphingomyelins, and phosphatidylcholines independently discriminate patients with PAH from control subjects and predict survival.
- Correction of metabolite levels over time is linked to better clinical outcomes, and patients who respond well to calcium channel blocker therapy have metabolic profiles comparable to those of healthy control subjects.

### What Are the Clinical Implications?

- Energy metabolism and stress-response pathways are disturbed in PAH.
- Monitoring plasma metabolites that report on these pathways over time could be useful to assess disease progression and response to therapy.
- Therapeutic strategies targeted against metabolic disturbances in PAH, particularly translational regulation and energy metabolism, merit further investigation.
