# Supplementary material for: Plasma Metabolomics Implicates Modified Transfer RNAs and Altered Bioenergetics in the Outcomes of Pulmonary Arterial Hypertension
Source: Circulation. 2017 Jan 30;135(5):460–75. doi: 10.1161/CIRCULATIONAHA.116.024602 (PMC5287439; doi:10.1161/CIRCULATIONAHA.116.024602)
Supplement: Supplementary file 1 [file cir-135-460-s001.pdf]

SUPPLEMENTAL MATERIAL

Title: Plasma metabolomics implicate modified transfer RNAs and altered bioenergetics in the outcome of pulmonary arterial hypertension

Authors: Christopher J. Rhodes[1]†, PhD; Pavandeep Ghataorhe[1]†, BM BCh; John Wharton[1], PhD; Kevin C. Rue-Albrecht[1], PhD; Charaka Hadinnapola[2], BM BCh; Geoffrey Watson[1], BM BCh; Marta Bleda[2], PhD; Matthias. Haimel[2], BSc; Gerry Coghlan[3], MD; Paul A. Corris[4], FRCP; Luke. S. Howard[5,6], DPhil; David G. Kiely[7,8], MD; Andrew J. Peacock[9], MD; Joanna Pepke-Zaba[10], PhD; Mark R. Toshner[2,10] , MD; S. John Wort[11], PhD; J. Simon R. Gibbs[5,6], MD; Allan Lawrie[8], PhD; Stefan Gräf[2,12], PhD; Nicholas W. Morrell[2], MD FMedSci;

Martin R. Wilkins[1*‡], MD FMedSci

Affiliations:

[1]Department of Medicine, Imperial College London, Hammersmith Campus, Du Cane Road, London, W12 0NN, UK

[2]Department of Medicine, University of Cambridge School of Clinical Medicine, Cambridge, CB2 OQQ, UK

[3]Cardiology Department, Royal Free Hospital, London, NW3 2QG, UK

[4]Institute of Cellular Medicine, Newcastle University and The Newcastle upon Tyne Hospitals NHS Foundation Trust, Newcastle upon Tyne, NE7 7DN, UK

[5]National Pulmonary Hypertension Service, Imperial College Healthcare NHS Trust, Hammersmith Hospital, Du Cane Road, London, W12 0HS, UK

[6]National Heart and Lung Institute (NHLI), Imperial College London, Hammersmith Campus, Du Cane Road, London, W12 0NN, UK

[7]Sheffield Pulmonary Vascular Disease Unit, Royal Hallamshire Hospital, Sheffield, S10 2JF, UK

[8]Department of Infection, Immunity & Cardiovascular Disease, University of Sheffield, Sheffield, S10 2RX, UK

[9]Scottish Pulmonary Vascular Unit, Golden Jubilee National Hospital, Glasgow, G81 4HX, UK

[10]Pulmonary Vascular Disease Unit, Papworth Hospital, Cambridge, CB23 3RE, UK

[11]Pulmonary Hypertension Service, Royal Brompton Hospital, London, SW3 6NP, UK

[12]Department of Haematology, University of Cambridge, Cambridge, CB2 0PT, UK

*Corresponding author: Dr Martin R Wilkins, MD FMedSci

Department of Medicine, Imperial College London, Hammersmith Campus, Du Cane Road, London, W12 0NN, UK.

Email: m.wilkins@imperial.ac.uk,

Tel: 020 3313 2049

†These authors contributed equally to this work

‡On behalf of the NIHR BioResource – Rare Diseases (BRIDGE) PAH Consortium and the UK National PAH Cohort Study Consortium

Supplementary Materials

Metabolomic profiling methodology

Samples were prepared with use of an automated MicroLab STAR system (Hamilton Company, Reno, NV, USA). For quality control (QC), a pooled sample from all experimental samples was used throughout the experiment, and a mixture of Metabolon QC standards were spiked into all experimental samples to monitor instrument performance and chromatographic alignment. Samples were randomised prior to experimentation.

Experiments were conducted on Waters Acuity ultra-performance liquid chromatography (UPLC) systems (Waters Corporation, Milford, MA, USA) using Thermo Scientific Q-Exactive high resolution/accurate mass spectrometer interfaced with a heated electrospray

ionization (HESI-II) source and Orbitrap mass analyser (Thermo Fisher Scientific, MA, USA).

The analysis platform used four methods for Ultrahigh Performance Liquid Chromatography-Tandem Mass Spectroscopy (UPLC-MS/MS) including a) positive ion mode electrospray ionisation (ESI), b) positive ion mode optimised for hydrophobic compounds, c) negative ion mode ESI and d) negative ionisation following elution from a hydrophilic interaction chromatography (HILIC) column. Scan time varied between methods and covered 70-1000m/z.

The resulting spectra were compared to the in-house Metabolon standard library using retention time, mass (m/z), adducts and MS/MS spectra. Analysis using this platform has been applied to measure metabolite levels in human plasma in control [1, 2] and disease populations [3, 4]. All experiments and runs were conducted on the same day.

Table S1 (below). Metabolites distinguishing pulmonary arterial hypertension (PAH) from healthy (HC) and disease controls (DC). 97 metabolites that are significantly different between PAH and healthy controls in 3 cohorts ($p<7.3e-5$) are shown. Mean values are given and the data is scaled to the healthy control group. Significance from linear regression is shown (p value), and for metabolites with $p>0.05$ in PAH HC linear regression, the significant confounder is shown. Significance is also shown for Mann Whitney U test between PAH treatment naïve patients versus all HC, and PAH bone morphogenetic protein type 2 receptor (*BMPR2*) mutation carriers versus patients with no *BMPR2* mutation. GPC, glycerophosphocholine. *probable metabolite identity, but unconfirmed (see methods).

Metabolite	Metabolic Pathway	Group averages (z-score relative to healthy control levels)									Statistics (p-values) and major confounders				
		Discovery			Validation1			Validation2	PAH	BMPR2	Linear Regression		Confounder	Sub-analyses	
		HC	DC	PAH (19-70)	HC	DC	PAH	PAH2	Naïve	Mutation	HC vs PAH	HC+DC vs PAH	HC vs PAH	PAH Naïve vs HC	BMPR2 vs PAH
Increased in PAH vs HC and DC (independent of confounders)															
N-acetylaspartate (NAA)	Alanine & Aspartate	0.12	0.21	1.13	-0.11	0.64	1.32	0.87	1.47	0.93	2.3E-04	4.6E-03		3.0E-09	0.46
octadecanedioate	Fatty Acid, Dicarboxylate	0.07	0.12	0.82	-0.07	0.34	0.93	0.35	0.95	0.43	1.5E-02	1.3E-02		7.3E-06	0.25
3-hydroxy-3-methylglutarate	Mevalonate	-0.07	0.57	0.89	0.07	0.57	1.15	0.76	1.20	0.75	3.5E-02	3.0E-02		1.6E-09	0.12
acisoga	Polyamine	-0.10	1.07	1.42	0.09	1.14	1.95	1.22	1.93	1.04	1.9E-04	1.5E-02		2.7E-11	2.9E-02
N1-methylinosine	Purine, Hypo-Xanthine/Inosine	-0.22	0.58	1.50	0.20	0.86	1.85	1.29	1.78	1.50	8.1E-04	2.2E-02		1.5E-13	0.48
xanthine	Purine, Hypo-Xanthine/Inosine	0.11	0.56	0.98	-0.10	0.62	1.06	1.00	1.35	1.16	2.0E-07	2.8E-03		3.4E-09	0.19
N2,N2-dimethylguanosine	Purine, Guanine	-0.11	0.83	1.56	0.10	1.15	2.00	1.39	1.95	1.36	1.1E-02	3.9E-02		2.2E-13	0.11
3-ureidopropionate	Pyrimidine, Uracil	-0.14	0.22	0.62	0.13	0.09	1.16	0.54	1.16	0.47	1.7E-02	9.0E-04		4.0E-09	0.36
malate	TCA Cycle	-0.18	0.60	1.17	0.17	0.69	1.64	1.02	1.81	1.11	8.8E-04	9.1E-03		1.5E-14	0.35
X - 12688	Unknown	-0.11	1.01	1.50	0.10	1.14	1.70	1.26	1.76	0.99	4.9E-05	3.2E-02		6.4E-13	1.6E-02
X - 13737	Unknown	-0.05	0.19	0.97	0.05	0.38	1.03	0.91	1.15	0.94	1.8E-02	6.1E-03		1.9E-08	0.70
X - 21796	Unknown	0.07	0.19	0.96	-0.07	0.44	1.06	0.73	1.33	0.83	5.0E-03	1.3E-02		1.9E-12	0.83
Decreased in PAH vs HC and DC (independent of confounders)															
palmitoylcholine	Fatty Acid (Acyl Choline)	-0.05	-0.14	-1.33	0.05	-0.35	-0.73	-1.10	-0.94	-1.35	1.7E-03	8.3E-03		3.9E-05	4.8E-02
1-arachidonoyl-GPC (20:4n6)*	Lysolipid	-0.08	-0.14	-1.09	0.08	-0.25	-0.74	-0.81	-0.81	-1.00	1.2E-02	3.2E-02		5.9E-05	0.27
1-docosapentaenoyl-GPC (22:5n3)*	Lysolipid	-0.03	-0.17	-1.03	0.03	-0.21	-0.80	-0.84	-0.79	-1.02	9.0E-03	6.7E-03		1.6E-05	0.23
1-linoleoyl-2-eicosapentaenoyl-GPC (18:2/20:5)*	Phospholipid	0.03	-0.39	-0.81	-0.03	-0.13	-1.13	-0.81	-1.16	-0.77	2.4E-02	1.2E-02		3.4E-08	0.68
sphingomyelin (d18:1/20:0, d16:1/22:0)*	Sphingolipid	0.05	-0.43	-1.02	-0.05	-0.38	-1.09	-1.05	-1.24	-0.93	1.3E-02	7.7E-03		1.2E-08	0.49
sphingomyelin (d18:1/21:0, d17:1/22:0, d16:1/23:0)*	Sphingolipid	0.03	-0.44	-0.89	-0.03	-0.32	-0.93	-0.76	-1.04	-0.83	2.7E-03	4.0E-02		1.4E-08	0.78
sphingomyelin (d18:1/22:1, d18:2/22:0, d16:1/24:1)*	Sphingolipid	0.07	-0.37	-1.09	-0.06	-0.32	-1.16	-1.03	-1.21	-1.12	2.6E-03	7.2E-04		1.8E-07	0.95
sphingomyelin (d18:2/23:0, d18:1/23:1, d17:1/24:1)*	Sphingolipid	0.04	-0.27	-0.63	-0.04	-0.17	-0.60	-0.54	-0.68	-0.66	4.9E-04	4.4E-02		1.0E-06	0.40

Increased in PAH vs HC (independent of confounders)														
oleoyl ethanolamide	Endocannabinoid	0.10	1.02	0.88	-0.09	1.15	1.02	0.52	0.84	0.29	1.3E-03	2.2E-01	5.6E-05	5.4E-03
3-hydroxybutyrylcarnitine (1)	Fatty Acid (Acyl Carnitine)	0.02	0.52	0.91	-0.01	0.81	0.97	0.60	1.11	0.62	6.1E-05	1.1E-01	7.3E-09	4.5E-02
3-hydroxybutyrylcarnitine (2)	Fatty Acid (Acyl Carnitine)	0.07	0.76	1.26	-0.06	1.02	1.24	0.90	1.49	0.81	4.7E-03	6.8E-01	3.4E-09	3.8E-02
acetylcarnitine	Fatty Acid (Acyl Carnitine)	0.11	0.43	1.02	-0.10	0.77	0.88	0.59	0.94	0.56	1.2E-02	2.9E-01	5.7E-06	3.1E-02
adipoylcarnitine	Fatty Acid (Acyl Carnitine)	-0.04	0.41	1.44	0.04	0.94	1.69	1.25	1.77	1.20	4.6E-02	3.9E-01	5.8E-10	0.09
myristoleoylcarnitine*	Fatty Acid (Acyl Carnitine)	0.04	0.33	0.75	-0.04	0.64	0.85	0.53	0.91	0.37	1.6E-02	4.6E-01	6.2E-05	1.0E-02
myristoylcarnitine	Fatty Acid (Acyl Carnitine)	0.01	0.46	0.81	-0.01	0.87	1.06	0.60	1.07	0.44	4.1E-02	9.5E-01	9.4E-06	1.1E-02
oleoylcarnitine	Fatty Acid (Acyl Carnitine)	0.03	0.53	0.96	-0.03	0.83	1.39	1.20	1.22	0.87	1.8E-02	6.7E-01	3.2E-07	0.10
palmitoleoylcarnitine*	Fatty Acid (Acyl Carnitine)	0.08	0.56	0.91	-0.07	0.90	1.23	0.85	1.14	0.69	8.5E-03	8.9E-01	7.9E-07	4.1E-02
suberoylcarnitine	Fatty Acid (Acyl Carnitine)	0.10	0.55	1.40	-0.09	0.98	1.61	1.23	1.66	1.03	7.1E-03	3.5E-01	1.3E-09	2.9E-02
glutamate	Glutamate	0.16	0.47	1.13	-0.14	0.41	0.98	0.71	1.12	0.83	2.9E-02	1.4E-01	2.7E-07	0.98
methionine sulfone	Met, Cys, SAM & Tau	-0.03	0.49	1.57	0.03	0.98	1.79	1.40	1.56	1.29	3.6E-02	4.9E-01	1.8E-09	0.14
N-acetylmethionine	Met, Cys, SAM & Tau	-0.04	0.48	1.08	0.04	0.63	1.27	0.60	1.30	0.80	3.3E-03	1.6E-01	1.3E-09	0.22
N-acetyltaurine	Met, Cys, SAM & Tau	0.07	0.56	1.02	-0.06	0.71	1.15	0.63	1.15	0.37	1.0E-03	7.1E-02	2.7E-07	9.7E-04
N-formylmethionine	Met, Cys, SAM & Tau	-0.17	0.49	1.08	0.16	0.69	1.53	0.78	1.59	0.85	4.8E-02	3.0E-01	5.8E-10	0.10
5,6-dihydrothymine	Pyrimidine, Thymine	-0.22	0.65	1.33	0.20	0.96	1.37	0.62	1.38	0.71	2.0E-04	6.7E-02	1.2E-08	0.15
alpha-ketoglutarate	TCA Cycle	-0.04	0.45	0.68	0.04	0.71	1.57	1.13	1.61	1.14	2.2E-02	2.4E-01	2.6E-13	1.00
fumarate	TCA Cycle	-0.15	0.30	0.65	0.13	0.45	1.14	0.56	1.23	0.31	2.1E-02	9.8E-02	3.5E-16	0.28
C-glycosyltryptophan	Tryptophan	-0.05	0.55	1.11	0.05	0.84	1.37	0.76	1.30	0.59	4.8E-02	3.9E-01	5.9E-07	4.7E-03
X - 12127	Unknown	0.06	0.63	0.85	-0.06	0.61	1.05	0.67	1.07	0.73	1.6E-02	4.4E-01	1.6E-08	0.53
X - 12472	Unknown	-0.04	0.71	1.03	0.04	0.82	1.05	0.96	1.15	0.93	3.4E-03	8.2E-01	2.5E-07	0.59
X - 12739	Unknown	-0.09	0.87	1.02	0.08	1.11	1.12	0.99	1.18	0.97	2.4E-02	1.8E-01	6.8E-07	0.35
X - 12824	Unknown	-0.02	0.54	0.85	0.02	0.74	1.08	0.79	1.16	0.78	2.0E-02	6.8E-01	9.5E-08	0.44
X - 17327	Unknown	0.03	0.66	0.86	-0.03	0.81	0.97	0.82	1.02	0.78	2.6E-02	7.0E-01	2.4E-06	0.34
X - 21829	Unknown	-0.07	0.44	0.82	0.06	0.65	1.24	0.66	1.41	0.62	2.5E-02	5.6E-01	2.4E-10	0.10
X - 24307	Unknown	0.04	0.68	0.70	-0.04	0.52	1.18	0.97	1.09	0.94	6.9E-05	7.2E-02	1.2E-07	0.88

X - 24513	Unknown	-0.10	0.67	1.05	0.09	0.92	1.44	0.98	1.30	0.77	2.4E-02	4.8E-01		1.4E-07	9.1E-03
X - 24527	Unknown	-0.08	0.86	1.11	0.08	1.17	1.22	1.06	1.22	0.94	3.5E-02	1.9E-01		2.2E-06	0.25
X - 24678	Unknown	0.21	0.74	1.20	-0.19	0.62	1.34	0.70	1.33	0.83	4.9E-03	1.2E-01		3.6E-11	0.42
X - 24766	Unknown	-0.31	0.45	0.82	0.28	0.60	1.07	0.85	0.89	0.92	1.9E-02	3.9E-01		5.1E-07	0.78
Decreased in PAH vs HC (independent of confounders)															
asparagine	Alanine & Aspartate	-0.06	-0.58	-0.88	0.05	-0.55	-1.00	-0.90	-1.22	-0.95	8.7E-04	8.5E-02		2.3E-07	0.64
dehydroisoandrosterone sulfate (DHEA-S)	Steroid	-0.05	-1.17	-1.53	0.04	-0.95	-1.74	-1.77	-1.53	-1.56	2.5E-02	4.1E-01		1.3E-09	0.46
X - 23765	Unknown	0.09	-0.50	-0.72	-0.08	-0.50	-0.90	-0.90	-1.02	-1.08	8.1E-04	2.0E-01		9.7E-07	0.15
Increased in PAH vs HC															
N-acetylalanine	Alanine & Aspartate	-0.11	0.55	0.76	0.10	0.88	1.15	0.66	1.04	0.50	9.9E-01	2.3E-01	Diuretics	3.2E-05	1.6E-02
N-acetylneuraminate	Aminosugar	-0.11	0.50	0.93	0.11	0.63	0.88	0.47	0.76	0.28	7.4E-02	2.4E-01	Age	1.8E-04	1.0E-03
erythronate*	Aminosugar	-0.11	0.45	0.71	0.10	0.68	1.16	0.59	1.10	0.37	7.1E-01	6.5E-01	Bilirubin	5.4E-06	6.0E-03
N-acetylglucosamine/N-acetylgalactosamine	Aminosugar	-0.14	0.50	0.72	0.13	0.89	0.95	0.58	0.62	0.54	1.0E-01	7.3E-01	Gender	1.8E-03	4.4E-02
gulonic acid*	Ascorbate & Aldarate	-0.08	0.49	0.61	0.07	0.57	0.79	0.61	0.81	0.47	8.5E-01	5.3E-01	Age	4.5E-05	2.8E-02
palmitoylcarnitine	Fatty Acid (Acyl Carnitine)	-0.03	0.49	0.91	0.03	1.03	1.28	0.95	1.23	0.90	6.0E-02	9.2E-01	Bilirubin	1.6E-07	0.43
malonylcarnitine	Fatty Acid Synthesis	0.00	0.20	0.56	0.00	0.29	0.94	0.48	0.96	0.57	1.3E-01	3.8E-02	Bilirubin	3.8E-09	0.53
N-acetylserine	Glycine, Serine & Threonine	-0.16	0.63	1.00	0.15	1.04	1.49	0.97	1.38	0.85	5.2E-01	4.0E-01	Bilirubin	6.9E-08	0.05
N-acetylthreonine	Glycine, Serine & Threonine	-0.22	0.31	0.68	0.20	0.64	0.99	0.55	1.10	0.47	6.5E-01	9.7E-01	Bilirubin	4.4E-07	2.0E-02
1-methylimidazoleacetate	Histidine	-0.05	0.52	0.86	0.04	0.50	1.32	0.86	1.02	0.74	6.0E-02	9.5E-02	Age	7.9E-07	0.08
imidazole propionate	Histidine	-0.21	0.44	0.84	0.19	0.71	0.99	0.79	0.98	0.84	1.4E-01	8.1E-01	Bilirubin	1.1E-07	0.54
quinolinate	Nicotinate & Nicotinamide	-0.11	0.46	0.76	0.10	0.68	1.19	0.86	0.95	0.58	6.0E-01	5.0E-01	Bilirubin	1.8E-04	3.8E-02
vanillylmandelate (VMA)	Phenylalanine & Tyrosine	-0.21	0.35	1.00	0.19	0.37	1.52	0.83	1.75	0.96	2.3E-01	1.5E-02	Age	6.3E-12	0.37
4-acetamidobutanoate	Polyamine	-0.10	0.72	1.22	0.09	0.90	1.93	1.26	1.83	1.19	8.6E-01	7.0E-01	Bilirubin	1.0E-11	0.15
N-acetylputrescine	Polyamine	-0.16	0.66	0.84	0.15	0.72	1.32	0.66	0.93	0.53	5.8E-01	6.2E-01	Bilirubin	3.0E-06	3.8E-02
N6-carbamoylthreonyl adenosine	Purine, Adenine	-0.12	0.58	1.04	0.11	0.90	1.49	1.06	1.31	0.92	1.7E-01	4.5E-01	Age	8.4E-09	4.6E-02
N1-methyladenosine	Purine, Adenine	-0.21	0.70	0.94	0.19	0.91	1.26	0.65	1.03	0.46	8.6E-01	1.4E-01	Bilirubin	2.4E-05	4.4E-03
N6-succinyladenosine	Purine, Adenine	-0.06	0.38	0.63	0.05	0.43	0.86	0.50	0.94	0.44	1.5E-01	2.7E-01	Bilirubin	1.2E-09	2.7E-03
N4-acetylcytidine	Pyrimidine, Cytidine	0.07	0.55	1.31	-0.06	0.77	1.50	1.22	1.33	1.19	2.7E-01	6.7E-01	Bilirubin	1.3E-08	0.47
orotidine	Pyrimidine, Orotate	-0.11	0.58	1.07	0.10	0.95	1.27	0.96	1.27	0.83	2.8E-01	5.3E-01	Bilirubin	1.4E-08	0.08
pseudouridine	Pyrimidine, Uracil	-0.08	0.77	1.22	0.08	1.05	1.66	1.17	1.68	0.97	1.5E-01	8.6E-01	Bilirubin	5.2E-11	1.4E-02

kynurenine	Tryptophan	-0.14	0.29	1.00	0.13	0.57	1.37	0.77	1.44	0.68	5.6E-01	3.3E-01	Bilirubin	1.2E-08	0.07
X - 12100	Unknown	-0.13	0.49	1.04	0.12	0.85	1.26	0.88	1.28	0.75	1.3E-01	4.6E-01	Age	9.8E-08	0.06
X - 11564	Unknown	-0.13	0.51	0.94	0.12	0.90	1.48	0.83	1.43	0.73	9.6E-01	8.8E-01	Bilirubin	3.5E-07	0.06
X - 12026	Unknown	-0.16	0.69	1.58	0.14	1.01	2.01	1.36	2.20	1.26	6.9E-01	7.7E-01	Bilirubin	3.8E-12	0.07
X - 12117	Unknown	-0.12	0.66	1.18	0.11	0.98	1.55	1.35	1.50	1.20	6.7E-01	3.6E-01	Bilirubin	7.7E-09	0.19
X - 15503	Unknown	-0.17	0.28	1.04	0.15	0.70	1.64	1.06	1.68	1.33	6.3E-01	5.6E-02	Bilirubin	2.5E-10	0.63
X - 11429	Unknown	-0.07	0.88	1.52	0.07	1.31	1.98	1.45	1.88	1.18	9.1E-02	8.4E-01	Diuretics	3.4E-10	2.0E-02
X - 21736	Unknown	-0.06	0.33	1.04	0.06	0.59	1.46	0.92	1.57	0.95	2.7E-01	3.5E-01	Diuretics	1.1E-10	0.42
pro-hydroxy-pro	Urea cycle; Arginine & Proline	-0.03	0.32	0.87	0.03	0.51	1.24	0.90	1.07	0.98	9.6E-01	6.9E-01	Diuretics	2.9E-06	0.95
Decreased in PAH vs HC															
histidine	Histidine	-0.07	-0.74	-1.57	0.06	-0.91	-1.89	-1.71	-1.78	-1.60	6.1E-02	8.2E-03	Prostanoids	1.2E-11	4.2E-01
1-linoleoyl-GPC (18:2)	Lysolipid	-0.10	-0.59	-1.22	0.09	-0.51	-1.14	-1.12	-1.33	-1.10	5.8E-02	2.4E-02	Bilirubin	1.3E-07	5.2E-01
2-linoleoyl-GPC (18:2)*	Lysolipid	-0.11	-0.55	-1.23	0.10	-0.42	-0.97	-1.09	-1.35	-1.15	9.4E-02	2.9E-02	Bilirubin	4.5E-08	9.6E-01
1-dihomo-linoleoyl-GPC (20:2)*	Lysolipid	0.00	-0.39	-0.95	0.00	-0.49	-0.86	-1.14	-0.98	-1.30	2.4E-01	4.3E-01	Statins	5.5E-05	1.2E-01
1-(1-enyl-palmitoyl)-2-linoleoyl-GPC (P-16:0/18:2)*	Plasmalogen	0.01	-0.38	-1.06	-0.01	-0.17	-1.13	-0.98	-1.28	-1.08	2.3E-01	1.5E-01	PDE5 inhib	5.1E-08	9.4E-01
behenoyl sphingomyelin (d18:1/22:0)*	Sphingolipid	0.02	-0.22	-0.75	-0.01	-0.20	-0.71	-0.92	-0.78	-0.77	1.4E-01	3.0E-02	Gender	3.6E-06	8.4E-01
4-androsten-3beta,17beta-diol disulfate (1)	Steroid	-0.04	-0.64	-0.87	0.03	-0.47	-0.81	-1.14	-0.55	-1.06	2.4E-01	5.4E-01	Gender	4.0E-03	6.8E-01
4-androsten-3beta,17beta-diol monosulfate (1)	Steroid	-0.01	-0.85	-1.15	0.01	-0.68	-1.30	-1.40	-1.18	-1.32	5.6E-02	3.7E-01	Gender	5.8E-07	9.3E-01
androsterone sulfate	Steroid	-0.02	-0.70	-1.33	0.02	-0.69	-1.40	-1.40	-1.14	-1.33	1.5E-01	2.0E-01	Gender	4.6E-06	7.3E-01
epiandrosterone sulfate	Steroid	-0.02	-0.86	-1.42	0.01	-0.72	-1.50	-1.49	-1.23	-1.40	1.3E-01	3.9E-01	Gender	1.1E-06	6.4E-01
pregn steroid monosulfate*	Steroid	-0.08	-0.83	-0.88	0.07	-0.68	-0.93	-1.04	-0.71	-0.84	1.0E-01	6.7E-01	Gender	3.3E-04	2.6E-01
X - 23749	Unknown	0.04	-0.16	-1.10	-0.03	-0.34	-1.05	-1.12	-0.94	-1.41	2.0E-01	2.7E-01	BMI	5.4E-05	0.02
arginine	Urea cycle; Arginine & Proline	-0.05	-0.67	-1.05	0.04	-0.79	-1.40	-1.47	-1.80	-1.32	1.1E-01	3.1E-01	DM drugs	2.1E-11	6.5E-01
homoarginine	Urea cycle; Arginine & Proline	-0.05	-0.70	-0.91	0.05	-0.59	-1.13	-1.06	-1.11	-0.86	5.1E-02	2.1E-01	Ethnicity	4.8E-07	7.6E-02

Table S1. Metabolites distinguishing pulmonary arterial hypertension (PAH) from healthy (HC) and disease controls (DC). 97 metabolites that are significantly different between PAH and healthy controls in 3 cohorts ($p<7.3e-5$) are shown. Mean values are given and the data is scaled to the healthy control group. Significance from linear regression is shown (p value), and for metabolites with $p>0.05$ in PAH HC linear regression, the significant confounder is shown. Significance is also shown for Mann Whitney U test between PAH treatment naïve patients versus all HC, and PAH bone morphogenetic protein type 2 receptor (*BMPR2*) mutation carriers versus patients with no *BMPR2* mutation. GPC, glycerophosphocholine. *probable metabolite identity, but unconfirmed (see methods).

Table S2. (below) Survival analysis.

Metabolite	Metabolic Pathway	Discovery		Validation1	
		Hazard Ratio	Sig	Hazard Ratio	Sig
Higher value indicates mortality. Independent of established prognostic markers					
N-acetylalanine	Alanine and Aspartate	2.02 (1.22-3.36)	6.43E-03	2.08 (1.12-3.86)	2.04E-02
pimeloylcarnitine/3-methyladipoylcarnitine	Fatty Acid (Acyl Carnitine)	2.16 (1.28-3.66)	4.03E-03	2.52 (1.24-5.10)	1.04E-02
1-methylimidazoleacetate	Histidine	2.26 (1.37-3.73)	1.43E-03	1.74 (1.03-2.93)	3.93E-02
N-acetylmethionine	Methionine, Cysteine, SAM and Taurine	2.36 (1.41-3.96)	1.16E-03	2.29 (1.18-4.43)	1.44E-02
N-formylmethionine	Methionine, Cysteine, SAM and Taurine	1.79 (1.20-2.68)	4.50E-03	1.98 (1.20-3.25)	7.15E-03
4-acetamidobutanoate	Polyamine	2.20 (1.45-3.35)	2.19E-04	2.02 (1.30-3.14)	1.83E-03
N-acetylputrescine	Polyamine	1.74 (1.04-2.91)	3.54E-02	2.92 (1.51-5.66)	1.50E-03
N1-methylinosine	Purine , (Hypo)Xanthine/Inosine	2.82 (1.74-4.57)	2.42E-05	1.73 (1.09-2.77)	2.11E-02
urate	Purine , (Hypo)Xanthine/Inosine	1.61 (1.06-2.42)	2.43E-02	2.14 (1.26-3.64)	4.73E-03
N6-succinyladenosine	Purine , Adenine	3.89 (1.40-10.82)	9.18E-03	8.31 (1.94-35.54)	4.29E-03
N6-carbamoylthreonyladenosine	Purine , Adenine	3.10 (1.60-6.00)	8.04E-04	2.08 (1.08-4.00)	2.81E-02
N1-methyladenosine	Purine , Adenine	1.94 (1.25-3.01)	2.92E-03	1.93 (1.12-3.32)	1.75E-02
N2,N2-dimethylguanosine	Purine , Guanine	2.53 (1.57-4.08)	1.35E-04	1.86 (1.14-3.03)	1.25E-02
pseudouridine	Pyrimidine , Uracil	1.78 (1.07-2.94)	2.54E-02	2.75 (1.48-5.12)	1.45E-03
X - 24020	Unknown	2.47 (1.42-4.30)	1.36E-03	1.84 (1.00-3.39)	4.94E-02
X - 24513	Unknown	2.34 (1.28-4.29)	5.92E-03	2.18 (1.11-4.29)	2.34E-02
X - 12472	Unknown	2.24 (1.44-3.47)	3.11E-04	1.57 (1.00-2.46)	4.97E-02
X - 12739	Unknown	2.07 (1.41-3.04)	2.07E-04	1.56 (1.06-2.28)	2.28E-02
X - 24527	Unknown	1.85 (1.38-2.48)	4.09E-05	1.43 (1.03-1.99)	3.12E-02
X - 12688	Unknown	1.81 (1.23-2.68)	2.83E-03	2.02 (1.27-3.19)	2.80E-03
X - 24728	Unknown	1.70 (1.09-2.64)	1.96E-02	2.04 (1.09-3.79)	2.50E-02
X - 15503	Unknown	1.67 (1.11-2.52)	1.38E-02	1.51 (1.03-2.22)	3.48E-02
X - 11564	Unknown	1.62 (1.10-2.38)	1.49E-02	1.60 (1.03-2.47)	3.51E-02

X - 24411	Unknown	1.54 (1.04-2.27)	3.05E-02	2.16 (1.32-3.54)	2.17E-03
X - 11429	Unknown	1.47 (1.05-2.06)	2.65E-02	1.79 (1.18-2.71)	5.76E-03
Lower value indicates mortality. Independent of established prognostic markers					
1-eicosapentaenoyl-GPE (20:5)*	Lysolipid	0.60 (0.43-0.84)	2.65E-03	0.62 (0.39-1.00)	4.98E-02
1-eicosapentaenoyl-GPC (20:5)*	Lysolipid	0.47 (0.31-0.73)	6.30E-04	0.50 (0.31-0.82)	6.05E-03
1-linoleoyl-2-docosahexaenoyl-GPC (18:2/22:6)*	Phospholipid	0.64 (0.46-0.89)	7.40E-03	0.65 (0.47-0.91)	1.27E-02
1-oleoyl-2-docosapentaenoyl-GPC (18:1/22:5n6)*	Phospholipid	0.64 (0.47-0.87)	4.11E-03	0.62 (0.43-0.90)	1.06E-02
1-myristoyl-2-arachidonoyl-GPC (14:0/20:4)*	Phospholipid	0.57 (0.39-0.83)	3.68E-03	0.68 (0.48-0.96)	2.66E-02
phosphatidylcholine (18:0/20:5, 16:0/22:5n6)*	Phospholipid	0.54 (0.37-0.77)	7.30E-04	0.42 (0.25-0.72)	1.44E-03
1-palmitoyl-2-eicosapentaenoyl-GPC (16:0/20:5)*	Phospholipid	0.53 (0.37-0.75)	4.11E-04	0.43 (0.26-0.74)	1.96E-03
1-linoleoyl-2-eicosapentaenoyl-GPC (18:2/20:5)*	Phospholipid	0.48 (0.33-0.70)	1.38E-04	0.60 (0.41-0.88)	8.59E-03
1-myristoyl-2-docosahexaenoyl-GPC (14:0/22:6)*	Phospholipid	0.47 (0.31-0.72)	4.84E-04	0.49 (0.29-0.81)	5.62E-03
dehydroisoandrosterone sulfate (DHEA-S)	Steroid	0.71 (0.52-0.97)	3.19E-02	0.55 (0.33-0.91)	2.06E-02
X - 24041	Unknown	0.46 (0.29-0.72)	6.88E-04	0.61 (0.38-0.99)	4.45E-02
Higher value indicates mortality					
hexadecanedioate	Fatty Acid, Dicarboxylate	1.53 (1.09-2.14)	1.40E-02	1.67 (1.16-2.41)	5.58E-03
N-acetyltaurine	Methionine, Cysteine, SAM and Taurine	1.97 (1.16-3.35)	1.19E-02	2.24 (1.13-4.43)	2.05E-02
3-hydroxy-3-methylglutarate	Mevalonate	1.68 (1.03-2.74)	3.78E-02	2.08 (1.02-4.23)	4.40E-02
vanillylmandelate (VMA)	Phenylalanine and Tyrosine	1.48 (1.06-2.06)	2.02E-02	1.58 (1.06-2.34)	2.38E-02
N-acetyl-beta-alanine	Pyrimidine , Uracil	1.54 (1.08-2.21)	1.80E-02	1.65 (1.18-2.32)	3.65E-03
fumarate	TCA Cycle	1.70 (1.00-2.88)	4.97E-02	2.57 (1.09-6.06)	3.09E-02
N2,N5-diacetylornithine	Urea cycle; Arginine and Proline	2.15 (1.27-3.63)	4.37E-03	2.15 (1.13-4.09)	1.95E-02
Lower value indicates mortality					
valine	Leucine, Isoleucine and Valine	0.66 (0.49-0.89)	6.80E-03	0.68 (0.48-0.95)	2.21E-02
1-docosahexaenoyl-GPE (22:6)*	Lysolipid	0.68 (0.49-0.94)	1.86E-02	0.54 (0.34-0.85)	8.27E-03
1-dihomo-linolenoyl-GPC (20:3n3 or 6)*	Lysolipid	0.57 (0.41-0.79)	7.43E-04	0.71 (0.53-0.96)	2.82E-02
1-docosahexaenoyl-GPC (22:6)*	Lysolipid	0.54 (0.35-0.81)	3.17E-03	0.57 (0.35-0.93)	2.32E-02
phosphatidylcholine (16:0/22:5n3, 18:1/20:4)*	Phospholipid	0.68 (0.48-0.96)	2.64E-02	0.59 (0.36-0.98)	4.16E-02
1-stearoyl-2-docosahexaenoyl-GPE (18:0/22:6)*	Phospholipid	0.64 (0.47-0.88)	5.99E-03	0.52 (0.32-0.85)	8.73E-03
1-palmitoleoyl-2-docosahexaenoyl-GPC (16:1/22:6)*	Phospholipid	0.62 (0.43-0.90)	1.09E-02	0.50 (0.28-0.88)	1.67E-02

1-pentadecanoyl-2-docosahexaenoyl-GPC (15:0/22:6)*	Phospholipid	0.62 (0.42-0.90)	1.30E-02	0.34 (0.17-0.68)	1.96E-03
1-stearoyl-2-docosahexaenoyl-GPC (18:0/22:6)	Phospholipid	0.60 (0.42-0.87)	7.16E-03	0.45 (0.27-0.76)	2.76E-03
1-stearoyl-2-docosapentaenoyl-GPC (18:0/22:5n3)*	Phospholipid	0.58 (0.41-0.82)	2.11E-03	0.50 (0.30-0.83)	8.18E-03
1-palmitoyl-2-dihomo-linolenoyl-GPC (16:0/20:3n3 or 6)*	Phospholipid	0.58 (0.38-0.88)	9.67E-03	0.54 (0.35-0.84)	6.47E-03
1-(1-enyl-stearoyl)-2-docosahexaenoyl-GPC (P-18:0/22:6)*	Plasmalogen	0.56 (0.37-0.85)	6.20E-03	0.57 (0.33-0.98)	4.06E-02
1-(1-enyl-stearoyl)-2-docosahexaenoyl-GPE (P-18:0/22:6)*	Plasmalogen	0.55 (0.37-0.82)	3.45E-03	0.49 (0.27-0.91)	2.31E-02
1-(1-enyl-palmitoyl)-2-docosahexaenoyl-GPC (P-16:0/22:6)*	Plasmalogen	0.54 (0.37-0.79)	1.31E-03	0.53 (0.32-0.87)	1.30E-02
1-(1-enyl-palmitoyl)-2-docosahexaenoyl-GPE (P-16:0/22:6)*	Plasmalogen	0.49 (0.34-0.71)	1.63E-04	0.47 (0.26-0.82)	8.30E-03
sphingomyelin (d18:2/14:0, d18:1/14:1)*	Sphingolipid	0.73 (0.53-1.00)	4.83E-02	0.48 (0.30-0.77)	2.36E-03
sphingomyelin (d18:1/20:0, d16:1/22:0)*	Sphingolipid	0.60 (0.41-0.87)	6.60E-03	0.57 (0.35-0.93)	2.32E-02
sphingomyelin (d18:1/22:1, d18:2/22:0, d16:1/24:1)*	Sphingolipid	0.59 (0.42-0.83)	2.51E-03	0.53 (0.33-0.85)	7.63E-03
behenoyl sphingomyelin (d18:1/22:0)*	Sphingolipid	0.42 (0.27-0.64)	5.94E-05	0.47 (0.27-0.82)	7.42E-03

Table S2. Survival analysis in PAH. 62 metabolites significantly different between PAH survivors and non-survivors in discovery and validation1 cohorts ($p<0.05$) are shown. Hazard ratio and significance (Sig) is shown from Cox regression analysis. Hazard ratios shown are for 1 standard deviation change in each metabolite for ease of comparison. Metabolites which are also independent of established prognostic markers in the discovery cohort are also shown. *probable metabolite identity, but unconfirmed (see methods). GPC, glycerophosphocholine; GPE, glycerophosphoethanolamine.

Pathway	Result	Metabolite	HC v PAH	Prog-nosis
Fatty Acid Metabolism(Acyl Carnitine)	4.5E-05	3-hydroxybutyrylcarnitine (1)	***6E-05***	0.3499
		pimeloylcarnitine/3-methyladipoylcarnitine	NA	***0.0006***
		suberoylcarnitine	***0.0071***	***0.0044***
		3-hydroxybutyrylcarnitine (2)	***0.0047***	***0.0046***
		palmitoleoylcarnitine*	***0.0085***	0.3042
		adipoylcarnitine	***0.0464***	***0.0106***
		acetylcarnitine	***0.0118***	0.141
		myristoleoylcarnitine*	***0.0165***	0.6142
		oleoylcarnitine	***0.0176***	0.0598
		myristoylcarnitine	***0.0412***	0.2631
		palmitoylcarnitine	0.0604	0.1959
		linoleoylcarnitine*	NA	0.0615
		hexanoylcarnitine	NA	0.5981
		stearoylcarnitine	NA	0.6611
		octanoylcarnitine	NA	0.8074
		laurylcarnitine	NA	0.8641
		decanoylcarnitine	NA	0.912
		cis-4-decenoyl carnitine	NA	0.9541
Polyamine Metabolism	0.01079	N-acetylputrescine	0.5825	***2E-05***
		4-acetamidobutanoate	0.8618	***3E-05***
		acisoga	***0.0002***	***0.0003***
		5-methylthioadenosine (MTA)	NA	***0.0433***
Alanine and Aspartate Metabolism	0.02412	N-acetylaspartate (NAA)	***0.0002***	0.2634
		asparagine	***0.0009***	0.9422
		N-acetylalanine	0.9901	***0.009***
		alanine	NA	0.6529
		aspartate	NA	0.9677
Purine Metabolism, (Hypo)Xanthine/Inosine containing	0.04317	xanthine	***2E-07***	0.438
		urate	NA	***0.0004***
		N1-methylinosine	***0.0008***	***0.0144***
		AICA ribonucleotide	NA	***0.0066***
		allantoin	NA	0.0934
		hypoxanthine	NA	0.6652
Purine Metabolism, Adenine containing	0.04317	N6-carbamoylthreonyladenosine	0.1735	***7E-05***
		N6-succinyladenosine	0.1452	***0.0005***
		N1-methyladenosine	0.8632	***0.0006***
		adenine	NA	0.4835
		adenosine	NA	0.5677
		adenosine 5'-monophosphate (AMP)	NA	0.5988
Pyrimidine Metabolism, Uracil containing	0.04317	pseudouridine	0.1506	***6E-05***
		N-acetyl-beta-alanine	NA	***0.0002***
		3-ureidopropionate	***0.0175***	***0.0024***
		uridine	NA	0.2684
		5-methyluridine (ribothymidine)	NA	0.5196
		beta-alanine	NA	0.8665

Table S3. Pathway enrichment analysis results. Pathways analysed and enrichment p-values are given, as well as metabolites within each pathway and significance values from tests used to select metabolites considered to be disease-associated. HC v PAH, significance of PAH on metabolite levels after controlling for potential confounders by linear regression; Prognosis, weakest significance of metabolite association with survival by Cox analysis in discovery or validation cohorts. *probable metabolite identity, but unconfirmed (see methods).

Metabolite	Area	Sig.	95% CI
3-hydroxy-3-methylglutarate	0.75	2.05E-04	0.63 - 0.86
3-hydroxybutyrylcarnitine (2)	0.67	1.10E-02	0.54 - 0.79
4-acetamidobutanoate	0.67	8.40E-03	0.56 - 0.79
5,6-dihydrothymine	0.67	1.04E-02	0.55 - 0.79
acetylcarnitine	0.65	2.80E-02	0.52 - 0.77
C-glycosyltryptophan	0.66	1.82E-02	0.53 - 0.78
fumarate	0.67	1.01E-02	0.55 - 0.79
hexadecanedioate	0.65	2.32E-02	0.52 - 0.78
malate	0.69	3.32E-03	0.58 - 0.81
N1-methyladenosine	0.69	3.62E-03	0.57 - 0.81
N2,N2-dimethylguanosine	0.69	4.70E-03	0.57 - 0.80
N-acetylalanine	0.67	1.01E-02	0.55 - 0.79
N-acetylmethionine	0.76	6.94E-05	0.66 - 0.87
N-acetylputrescine	0.69	4.07E-03	0.57 - 0.81
N-acetyltaurine	0.68	6.40E-03	0.56 - 0.80
N-formylmethionine	0.67	8.63E-03	0.56 - 0.79
X - 11564	0.65	2.16E-02	0.53 - 0.77
X - 12127	0.65	2.43E-02	0.53 - 0.77
X - 12472	0.65	2.32E-02	0.53 - 0.77
X - 12688	0.68	5.26E-03	0.57 - 0.80
X - 12739	0.68	7.75E-03	0.56 - 0.79
X - 13737	0.66	1.45E-02	0.54 - 0.78
X - 15503	0.68	5.89E-03	0.56 - 0.80
X - 21796	0.70	3.03E-03	0.59 - 0.80
X - 24020	0.67	1.15E-02	0.55 - 0.78
X - 24527	0.67	8.63E-03	0.55 - 0.79
X - 24766	0.67	1.07E-02	0.55 - 0.78

Table S4. ROC analysis of serial metabolite measurements. Area under the curve values for the association between metabolite level changes (i.e. sample 1 subtracted from sample 2) and survival during follow-up are shown for significantly associated metabolites. *probable metabolite identity, but unconfirmed (see methods).

	Controls n=30	PAH (19-70) n=69	PAH (>70) n=8
Female:Male ratio	2.0	2.3	3.0
Age	48.5 +/- 13.1	48.3 +/- 14.1	75.6 +/- 8.1
Angiogenin conc., ng/ml	360 +/- 110.3	479.7 +/- 176.6	554.9 +/- 234.2
N2,N2-dimethylguanosine	-0.1 +/- 1	1.6 +/- 1.2	2.7 +/- 1.4

Table S5. Demographics and circulating factor levels in subjects used for angiogenin study.

Mean +/- standard deviation is shown for continuous variables.

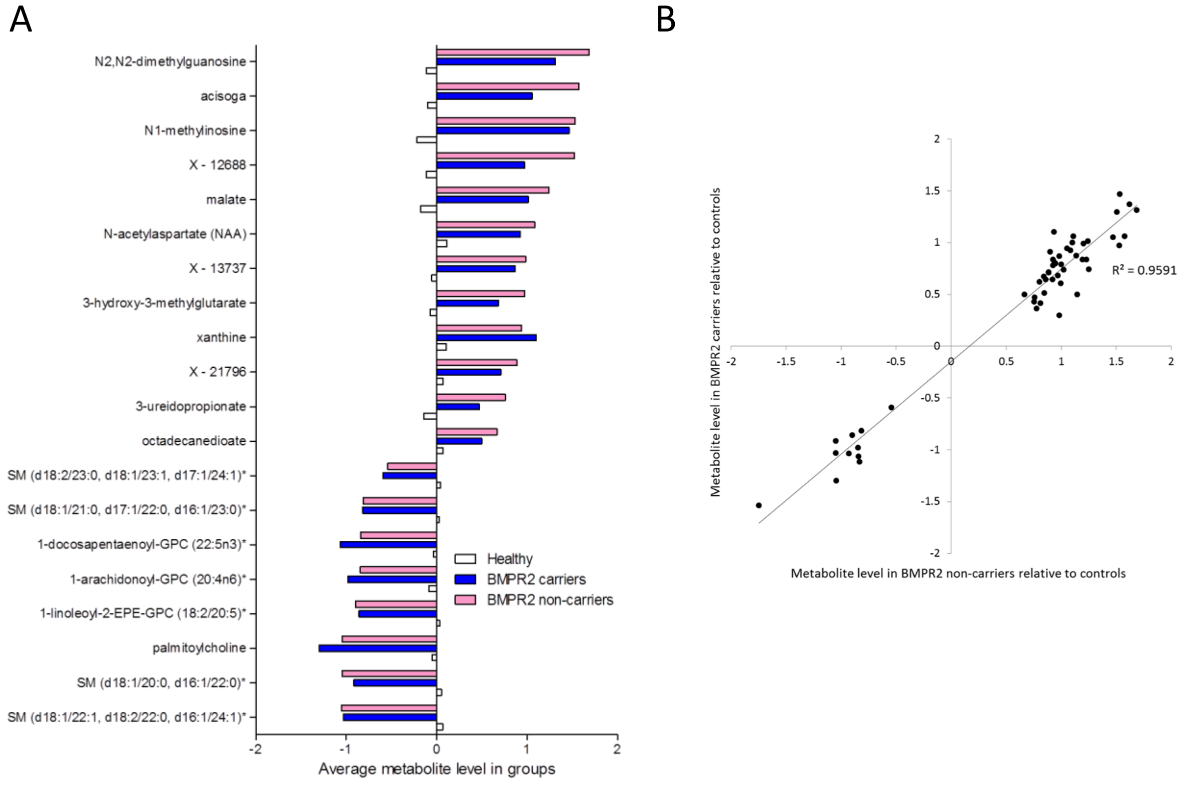

Fig. S1. Metabolite levels in BMRP2 mutation carriers. (**A**) Average metabolite levels in PAH BMPR2 mutation carriers, non-carriers and control subjects for 20 metabolites found to significantly distinguish PAH and both healthy and disease controls, independent of potential confounders. (**B**) Correlation of average metabolite levels in BMPR2 mutation carriers and non-carriers relative to controls for 53 metabolites that distinguish PAH from healthy controls, independent of potential

confounders. Values plotted are z-scores calculated based on mean and standard deviation of all healthy volunteers in study - negative values indicate metabolites at lower levels in patients versus healthy controls and positive values indicate higher levels of metabolites in patients. *probable metabolite identity, but unconfirmed (see methods).

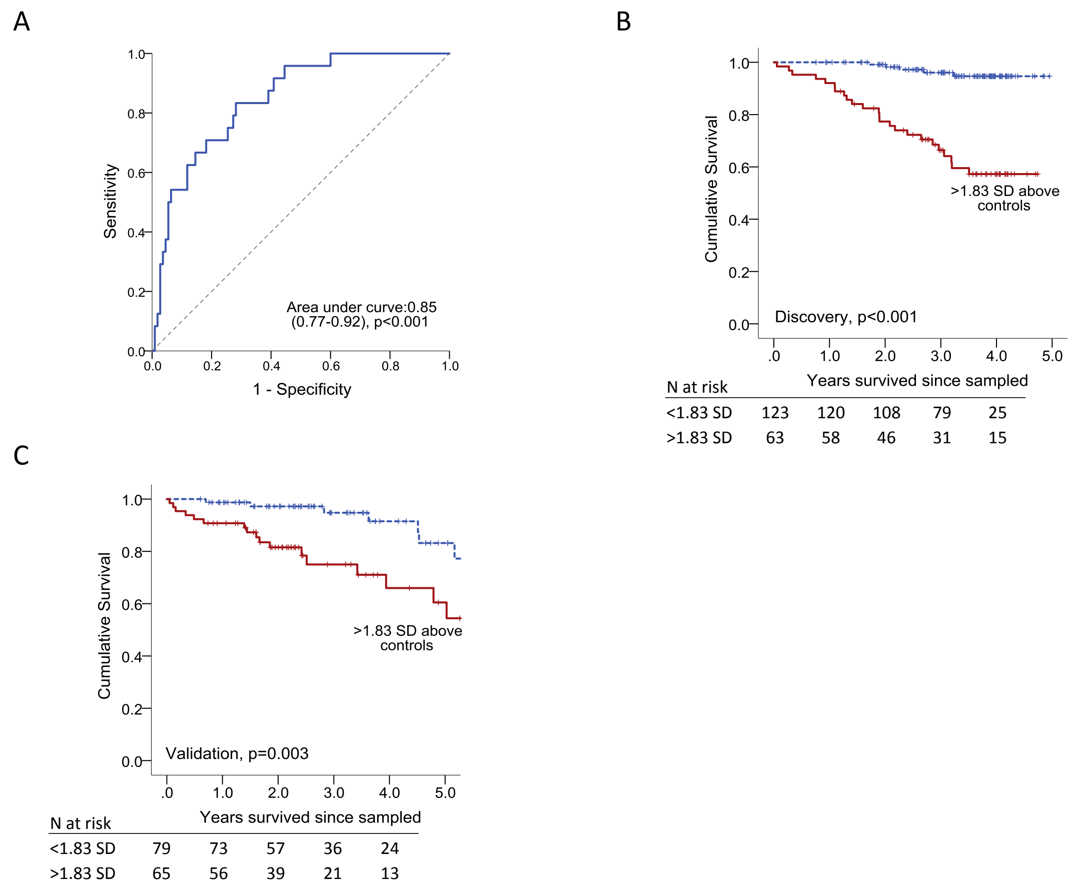

Fig. S2– Survival analysis of PAH patients. (**A**) Receiver operating characteristic (ROC) curve for N2,N2-dimethylguanosine in the discovery cohort at 3 years of follow-up. The optimal cut-off for high/low risk levels of N2,N2-dimethylguanosine was derived from this for **B&C.** Kaplan Meier survival estimates in PAH patients in the discovery (**B**) and first validation (**C**) cohorts.

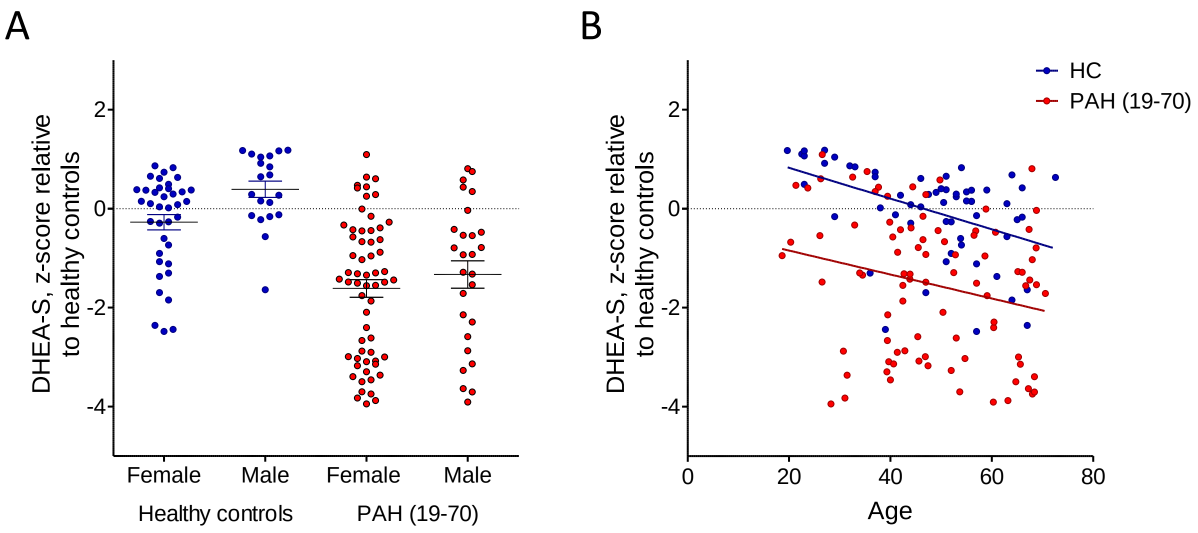

Fig. S3. Dehydroisoandrosterone-sulphate (DHEA-S). Plasma DHEA-S levels in the discovery cohort are shown for healthy controls (HC) and PAH (19-70) with (**A**) separation by gender and (**B**) against age.

Supplementary References

1. Shin SY, Fauman EB, Petersen AK, Krumsiek J, Santos R, Huang J, Arnold M, Erte I, Forgetta V, Yang TP, Walter K, Menni C, Chen L, Vasquez L, Valdes AM, Hyde CL, Wang V, Ziemek D, Roberts P, Xi L, Grundberg E, Multiple Tissue Human Expression Resource C, Waldenberger M, Richards JB, Mohney RP, Milburn MV, John SL, Trimmer J, Theis FJ, Overington JP, Suhre K, Brosnan MJ, Gieger C, Kastenmuller G, Spector TD and Soranzo N. An atlas of genetic influences on human blood metabolites. *Nature genetics*. 2014;46:543-50.
2. Guo L, Milburn MV, Ryals JA, Lonergan SC, Mitchell MW, Wulff JE, Alexander DC, Evans AM, Bridgewater B, Miller L, Gonzalez-Garay ML and Caskey CT. Plasma metabolomic profiles enhance precision medicine for volunteers of normal health. *Proceedings of the National Academy of Sciences of the United States of America*. 2015;112:E4901-10.
3. Lawton KA, Brown MV, Alexander D, Li Z, Wulff JE, Lawson R, Jaffa M, Milburn MV, Ryals JA, Bowser R, Cudkowicz ME, Berry JD and Northeast ALSC. Plasma metabolomic biomarker panel to distinguish patients with amyotrophic lateral sclerosis from disease mimics. *Amyotrophic Lateral Sclerosis and Frontotemporal Degeneration*. 2014;15:362-70.
4. Miller MJ, Kennedy AD, Eckhart AD, Burrage LC, Wulff JE, Miller LA, Milburn MV, Ryals JA, Beaudet AL, Sun Q, Sutton VR and Elsea SH. Untargeted metabolomic analysis for the clinical screening of inborn errors of metabolism. *Journal of Inherited Metabolic Disease*. 2015;38:1029-39.
